# Overexpression of cyclooxygenase-2 in adipocytes reduces fat accumulation in inguinal white adipose tissue and hepatic steatosis in high-fat fed mice

**DOI:** 10.1038/s41598-019-45062-w

**Published:** 2019-06-20

**Authors:** Niels Banhos Danneskiold-Samsøe, Si Brask Sonne, Jeppe Madura Larsen, Ann Normann Hansen, Even Fjære, Marie Sophie Isidor, Sidsel Petersen, Jeanette Henningsen, Ilenia Severi, Loris Sartini, Yvonne Schober, Jacqueline Wolf, W. Andreas Nockher, Christian Wolfrum, Saverio Cinti, Christian Sina, Jacob B. Hansen, Lise Madsen, Susanne Brix, Karsten Kristiansen

**Affiliations:** 1 Laboratory of Genomics and Molecular Biomedicine, Department of Biology, University of Copenhagen, Universitetsparken 13, DK-2100 Copenhagen, Denmark; 2 National Food Institute, Technical University of Denmark, DK-2800 Kgs., Lyngby, Denmark; 3 Institute of Marine Research, P.O. Box 7800, 5020 Bergen, Norway; 4 School of Medicine, Department of Experimental and Clinical Medicine, Division of Neuroscience and Cell Biology, Università Politecnica delle Marche, via Tronto 10/A, 60020 Ancona, Italy; 5 Institute of Laboratory Medicine and Pathobiochemistry, Molecular Diagnostics, Philipps University Marburg, University Hospital Giessen and Marburg, Campus Marburg, Baldingerstrasse, 35043 Marburg, Germany; 6 Institute of Food Nutrition and Health, ETH Zürich, SLA C94, Schorenstrasse 16, CH-8603 Schwerzenbach, Switzerland; 7 Institute of Nutritional Medicine, Department of Internal Medicine I, University Hospital of Schleswig-Holstein, Ratzeburger Allee 160, 23538 Lübeck, Germany; 8 Department of Biotechnology and Biomedicine, Technical University of Denmark, DK-2800, Kgs. Lyngby, Denmark; 9 Institute of Metagenomics, BGI-Shenzhen, BGI-Shenzhen, Shenzhen, 518083 China

**Keywords:** Cell biology, Endocrinology, Immunology

## Abstract

Cyclooxygenases are known as important regulators of metabolism and immune processes via conversion of C20 fatty acids into various regulatory lipid mediators, and cyclooxygenase activity has been implicated in browning of white adipose tissues. We generated transgenic (TG) C57BL/6 mice expressing the *Ptgs2* gene encoding cyclooxygenase-2 (COX-2) in mature adipocytes. TG mice fed a high-fat diet displayed marginally lower weight gain with less hepatic steatosis and a slight improvement in insulin sensitivity, but no difference in glucose tolerance. Compared to littermate wildtype mice, TG mice selectively reduced inguinal white adipose tissue (iWAT) mass and fat cell size, whereas the epididymal (eWAT) fat depot remained unchanged. The changes in iWAT were accompanied by increased levels of specific COX-derived lipid mediators and increased mRNA levels of interleukin-33, interleukin-4 and arginase-1, but not increased expression of uncoupling protein 1 or increased energy expenditure. Epididymal WAT (eWAT) in TG mice exhibited few changes except from increased infiltration with eosinophils. Our findings suggest a role for COX-2-derived lipid mediators from adipocytes in mediating type 2 immunity cues in subcutaneous WAT associated with decreased hepatic steatosis, but with no accompanying induction of browning and increased energy expenditure.

## Introduction

Cyclooxygenases are essential for prostaglandin production by catalysing the first step in the biosynthesis of prostaglandins. At the mRNA level, cyclooxygenase-1 (COX-1) is constitutively expressed in adipocytes, whereas expression of cyclooxygenase-2 (COX-2) is induced in inguinal white adipose tissue (iWAT) in response to cold exposure concomitant with induction of uncoupling protein 1 (UCP1) expression^1,2^ or by administration of a β-adrenergic agonist^2^. Expression of COX-1, COX-2, and UCP1 is also upregulated by β-adrenergic stimulation of cultured adipocytes^1,2^. Injection of the stable prostaglandin E_2_ (PGE_2_) analogue 16,16-dimethyl-PGE_2_ increased expression of UCP1 in iWAT in C57BL/6 mice^1^. Furthermore, NMRI mice with overexpression of COX-2 in the skin exhibited markedly elevated levels of prostaglandins in circulation and had lower body weight and fat mass gain associated with increased energy expenditure, UCP1 expression, and thermogenesis^2^. Together these observations provided evidence for a link between cyclooxygenase activity, prostaglandins, and UCP1 expression.

High-fat diet (HFD) feeding of rats has been reported to increase expression of COX-1 and COX-2^3^. By contrast, in C57BL/6 mice HFD feeding decreased the level of COX-derived products suggesting that HFD feeding did not induce expression of COX in this mouse model^4^. These results were recently corroborated by a study demonstrating that up to 16 weeks of HFD feeding of C57BL/6 mice supressed COX-2 but not COX-1 expression in iWAT^5^.

Genetic or chemical modulation of cyclooxygenase activity in WAT has resulted in disparate effects on body composition and metabolism depending on the mouse model used. COX-2 knockout mice on a mixed C57BL/6J × 129/Ola background were reported to exhibit reduced body fat mass accumulation on a standard diet associated with increased oxygen consumption^6^, but interestingly these mice displayed equal body weight, but lower eWAT mass compared with wildtype (WT) mice on a high-fat/high-sucrose diet^7^. In these experiments UCP1 expression in adipose tissues was not examined^6,7^. COX-2 knockout mice on a mixed C57BL/6xSv129 background exhibited reduced cold-induced induction of Ucp1^1^, were cold sensitive, and less able to defend their body temperature compared to WT mice^1^. Similarly, COX-2 knockout mice on a mixed B6/129P2 background also exhibited impaired cold- and CL316,243-induced UCP1 expression in adipose tissues^2^.

Treatment with the general cyclooxygenase inhibitor indomethacin accentuated HFD-induced obesity in the obesity resistant Sv129 mice and reduced diet-induced expression of UCP1 in iWAT^1,8,9^. By contrast, supplementation with indomethacin reduced obesity development and improved insulin sensitivity in obesity prone HFD-fed C57BL/6J mice^8^, clearly pointing to mouse strain dependent differences in the responses to inhibition of cyclooxygenase activity.

Inhibition of COX activity has been reported to be associated with decreased expression of pro-inflammatory cytokines and chemokines including tumour necrosis factor α (*Tnfa*) and monocyte chemotactic protein-1 (*Ccl2*)^3–5^. Of note, anti-inflammatory cues in WAT have been associated with type 2 immune-mediated signalling, which has also been reported to play a role in browning, thermogenesis, and glucose metabolism in WAT^10–13^, contrasting the pro-inflammatory impairment of insulin sensitivity observed in obese mice^14^.

In WAT from rodents and in preadipocytes in culture, prostaglandins have been shown to regulate adipocyte differentiation both positively and negatively^15–23^, as well as to increase lipolysis^24,25^, increase release of adipokines^25^, and increase uncoupled respiration^1,2^. However, it is unclear if COX-2-derived lipid mediators produced in WAT contribute to type 2 immune processes.

In order to examine the effects of forced expression of COX-2 in adipose tissue of C57BL/6 mice, we generated transgenic mice with selective overexpression of COX-2 in mature adipocytes. We examined how this affected the level of COX-derived products in subcutaneous and visceral adipose tissue depots, and to what extend such overexpression affected *Ucp1* induction, systemic metabolic parameters, and local immune responses in HFD-fed mice.

## Results

### Overexpression of COX-2 in mature adipocytes leads to a marginally lower body weight gain in response to high fat feeding

**Figure 1:**
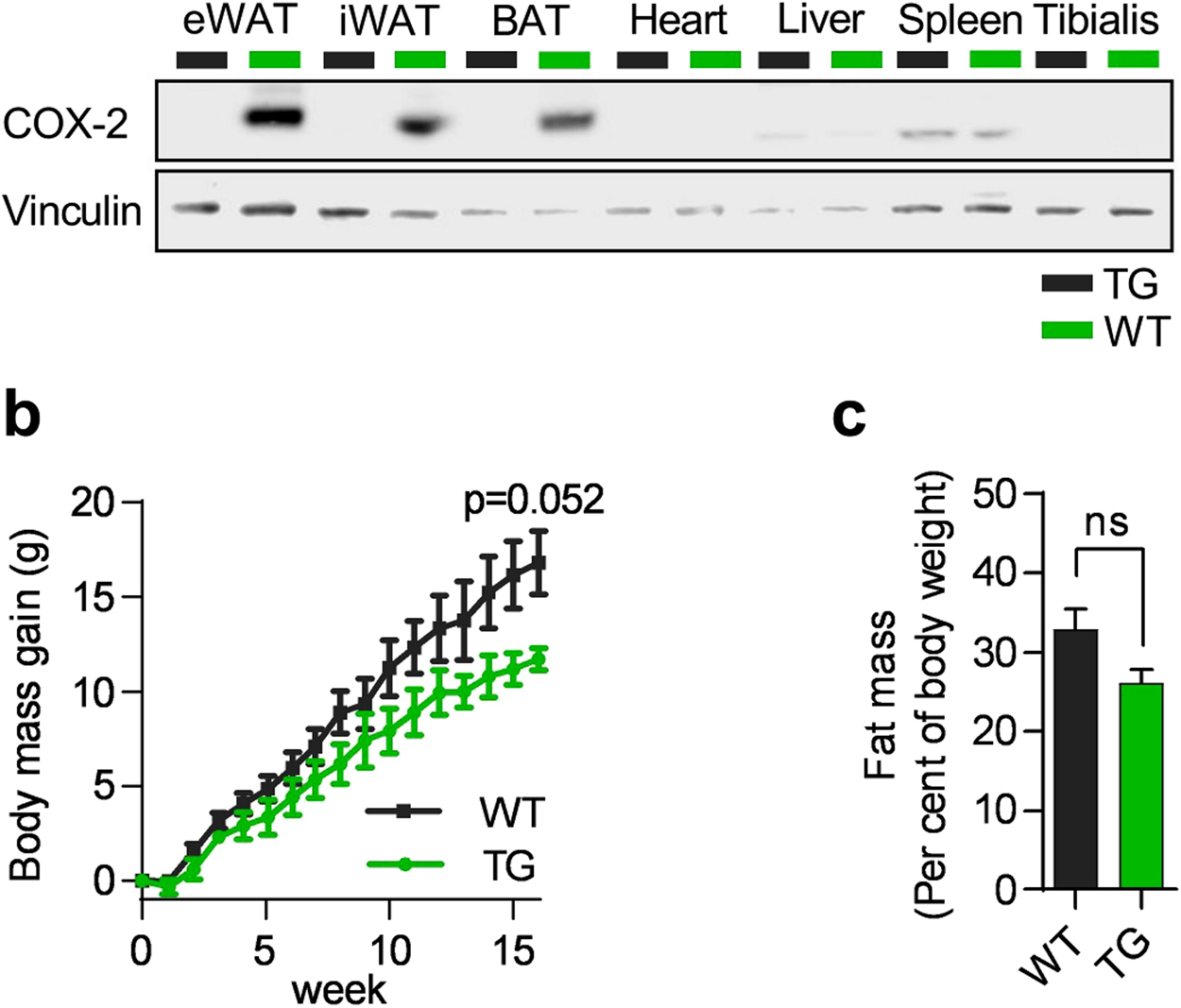
Overexpression of COX-2 in mature adipocytes leads to a marginally lower body weight gain in response to high fat feeding. (**a**) Western blot of COX-2 in wildtype (WT) and transgenic (TG) mice at thermoneutrality. Tibialis = Tibialis Anterior. PVDF membrane cut horizontally at 100 kDa and stained separately with antibodies. (**b**) Body weight gain on a HFD before adaptation to metabolic chambers. (**c**) Body fat mass in percent of body weight. Students t-test, ns represents nonsignificant and *P ≤ 0.05. Mean ± SEM.

We generated mice expressing *Ptgs2* under the control of a truncated adiponectin promoter^26^, leading to a strong induction of COX-2 expression in mature adipocytes of visceral eWAT, subcutaneous iWAT, and intrascapular brown adipose tissue (iBAT), but not in liver, heart, spleen or skeletal muscle (Fig. 1a). Overexpression of COX-2 has been shown to induce expression of cyclooxygenase 1 (*Ptgs1*) in adipocytes *in vitro*^1^, but in the TG mice forced expression of COX-2 did not alter mRNA levels of *Ptgs1* in WAT (Fig. S1a). No differences in body weight or total fat mass were found between TG and WT littermates before initiation of HF feeding (Fig. S1b,c). Feeding mice a HFD for 18 weeks led to marginally lower weight gain without differences in total fat mass in TG mice as compared to WT littermates (Fig. 1b,c), with no differences in apparent fat digestibility (Fig. S1d).

### Overexpression of COX-2 in mature adipocytes does not induce expression of *Ucp1* but reduces iWAT mass and alters adipocyte size

**Figure 2:**
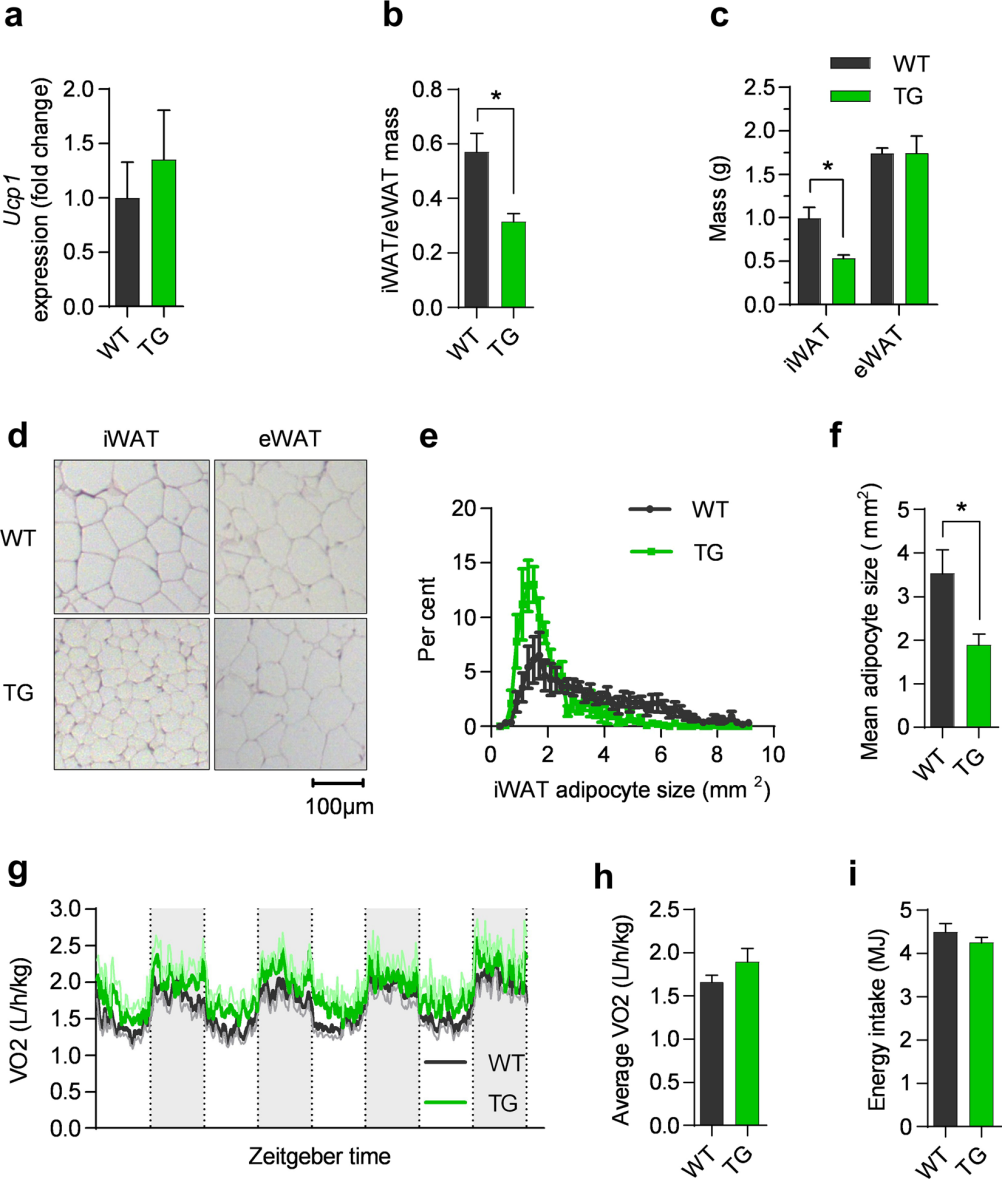
Overexpression of COX-2 in mature adipocytes does not induce expression of *Ucp1* but still reduces iWAT mass and alters adipocyte size. a-h:data from experiment 1 in mice on HFD for 18 weeks, i: data from experiment 4. (**a**) *Ucp1* mRNA in iWAT. (**b**) WT and TG iWAT/eWAT weight fraction. (**c**) iWAT and eWAT weight. (**d**) Representative H&E stains of iWAT and eWAT. (**e**) Histogram of mean adipocyte size, (n = 5–8). (**f**) Mean adipocyte size, (n = 5–8). (**g**) Oxygen consumption rate at thermoneutrality, error in light green and light gray. (**h**) Average oxygen consumption rate. (**i**) Accumulated energy intake (n = 7–8). Students t-test, ns represents nonsignificant and *P ≤ 0.05. Mean ± SEM.

Since COX-2 expression and activity have been linked with cold-induced UCP1 expression in WAT^1,2^, we investigated if expression of *Ucp1* was induced in WAT of the TG mice kept under thermoneutral conditions. Unexpectedly, no difference in *Ucp1* expression was found in iWAT (Fig. 2a), eWAT or BAT (Fig. S2a). Furthermore, expression of cell death activator CIDE-A (*Cidea*), peroxisome proliferator-activated receptor gamma coactivator 1-alpha (*Ppargc1a*), type II iodothyronine deiodinase (*Dio2*), and CCAAT/enhancer-binding protein beta (*Cebpb*) was not increased in the TG mice in iWAT or eWAT (Fig. S2c,d) while PR domain containing 16 (*Prdm16*) was decreased in eWAT of TG mice (Fig. S2c). Further, expression of *Ppargc1a*, *Cebpb*, and *Prdm16* in BAT was not increased in TG mice (Fig. S2d). Together, this indicated that COX-2 overexpression in mature adipocytes did not induce browning at thermoneutral conditions. However, we noticed that the relative weight of iWAT compared to eWAT was lower in TG mice compared to WT (Fig. 2B). This was due to lower iWAT mass, whereas eWAT mass was not altered by overexpression of COX-2 (Fig. 2c). Interestingly, feeding mice a HFD for three weeks was insufficient to induce differences in iWAT or eWAT mass in TG compared to WT mice (Fig. S2e). This indicates that substantial weight gain is needed for differences in expansion of these adipose depots, and that expansion is differentially affected in iWAT and eWAT. The weight and relative weight of BAT compared to eWAT was unaffected by overexpression of COX-2 (Fig. S2f,g). Due to the lower iWAT mass in TG mice, we examined adipose tissue morphology. Lower iWAT weight was associated with smaller adipocytes in iWAT of TG mice (Fig. 2d–f), but not in eWAT (Fig. 2d). Furthermore, also the morphology of BAT was unaffected by COX-2 overexpression (Fig. S2h). Thus, even though forced expression of COX-2 did not induce *Ucp1* expression, adipocyte mass and morphology was selectively affected in iWAT.

Mirroring the lack of *Ucp1* induction, we found no significant differences in oxygen consumption at week 18 as determined by indirect calorimetry or accumulated energy intake (Fig. 2g–i), and the comparable expression of glycerol kinase (*Gyk*) in WT and TG in iWAT and BAT (Fig. S2i) indicated that futile cycling of triglycerides was not upregulated. Furthermore, cold exposure in mice did not increase mRNA or protein expression of Ucp1 expression in TG mice compared to WT (Fig. S2j,k) indicating that β-adrenergic stimulation was insufficient to further increase uncoupled respiration in the TG mice. In support of this, respiration upon cold exposure of mice in metabolic chambers did not differ between WT and TG animals (Fig. S2l,m), demonstrating that β-adrenergic signalling was unable to initiate more pronounced adaptive thermogenesis in TG as compared to WT mice. Collectively, our data showed that decreased tissue mass and adipocyte size in iWAT of mice with overexpression of COX-2 in adipocytes were not associated with *Ucp1* expression and adaptive thermogenesis, and that futile cycling of triglycerides in WAT did not contribute to the decreased size of adipocytes in iWAT.

### Eicosanoids produced by COX-2 in iWAT and eWAT are selectively increased in TG mice

**Figure 3:**
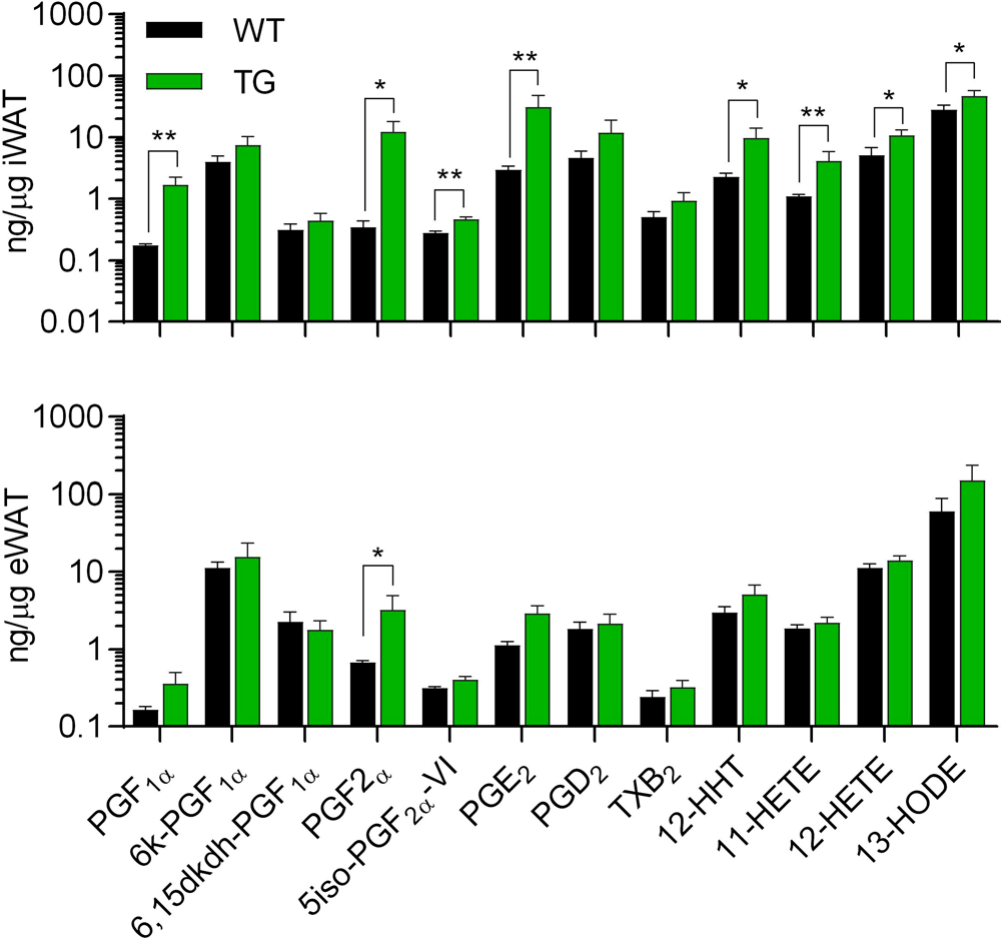
Eicosanoids produced by COX-2 in iWAT and eWAT are selectively increased in TG mice. Levels of COX-dependent and independent eicosanoids including PGF_1α_, 6-keto-PGF_1α_, 6.15dkdh-PGF_1α_, PGF_2α_, 5-iso-PGF_2α_-VI, PGE_2_, PGD_2_, TXB_2_, 12-HHT, 11-HETE, 12-HETE and 13-HODE in iWAT and eWAT after 18 weeks of HFD (experiment 1). Mann-Whitney U test for differences between groups with adjustment for false-discovery rate. * represents P ≤ 0.05 and **P ≤ 0.01.

Given the relatively modest changes in weight gain and fat mass, and lack of induction of *Ucp1* expression by overexpression of COX-2 in adipose tissues, we investigated to what extent overexpression of COX-2 affected the eicosanoid profiles in iWAT and eWAT by conducting a comprehensive analysis of COX-2-, lipoxygenase (LOX)-, and cytochrome P450-derived lipid metabolites (Table S2) in iWAT and eWAT. COX-2 overexpression led to markedly increased levels of classic COX-2-derived eicosanoids in iWAT of TG mice, including PGF_1α_, PGF_2α_, 5-iso-PGF_2α_-VI, PGE_2_ and putative prostanoids including 12-HHT, 12-HETE, 11-HETE and 13-HODE ranging from a 2-fold (5iso-PGF_2α_-VI) to a 20-fold (PGF_2α_) rise in concentration, while only PGF_2α_ was increased in eWAT (Fig. 3). Only minor changes were found among eicosanoids not dependent on COX-2 activity, except for an increased level of the LOX-derived 12-HEPE in iWAT (Fig. S3). In eWAT, 5oxo-ETE was the sole LOX-derived metabolite exhibiting increased levels (Figs 3 and S3). Taken together these results demonstrated that the overexpression of COX-2 differentially affected the eicosanoid profiles in eWAT and iWAT with the latter exhibiting the most pronounced increase in several COX-2-dependent eicosanoids, including PGE2 that previously has been reported to play a role in browning of iWAT in mice^1^ and in human adipocytes^27^.

### Expression of genes involved in adipocyte differentiation is differentially modulated in iWAT and eWAT of COX-2 overexpressing mice

**Figure 4:**
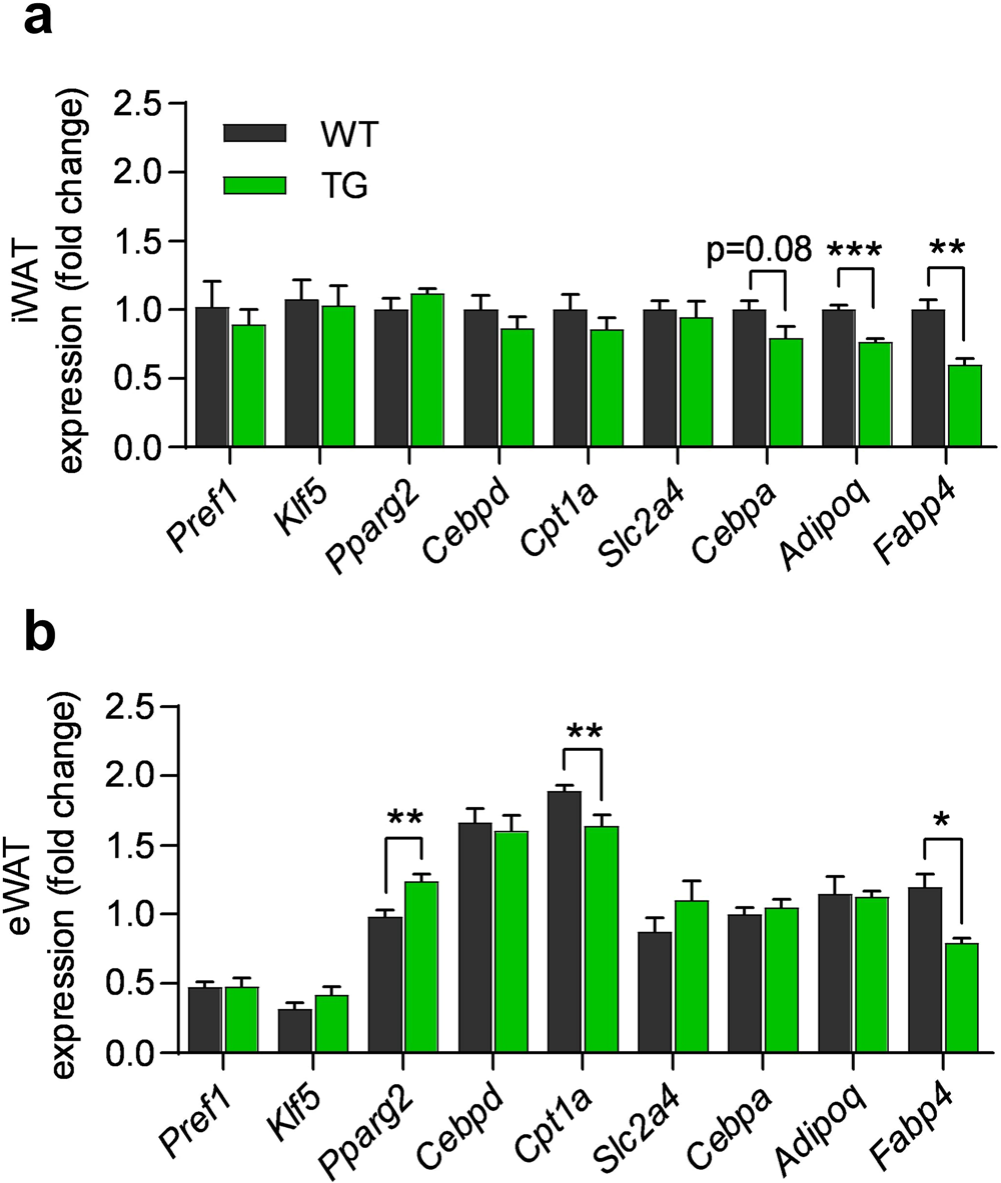
Expression of genes involved in adipocyte differentiation is differentially modulated in iWAT and eWAT of COX-2 overexpressing mice. (**a**) *Slc2a4*, *Pparg2*, *Cpt1a*, *Pref1*, *Klf5*, *Cebpa*, *Cebpd*, *Adipoq* and *Fabp4* mRNA expression in iWAT and (**b**) eWAT after 18 weeks on HFD (experiment 1). Expression normalised to WT in iWAT. Students t-test, ns represents nonsignificant, *P ≤ 0.05, **P ≤ 0.01 and ***P ≤ 0.001. Mean ± SEM.

Since we observed reduced adipocyte size selectively in iWAT from TG mice, we examined if expression of genes involved in lipid metabolism and adipogenesis was differentially altered in iWAT and eWAT (Fig. 4). We found reduced expression of fatty acid binding protein 4 (*Fabp4*) in both iWAT and eWAT of TG mice, while expression of other genes varied comparing iWAT and eWAT (Fig. 4). Notably, expression of the late adipogenic marker CCAAT/enhancer-binding protein alpha (*Cebpa*) was marginally reduced (p < 0.08) and adiponectin (*Adipoq*) was significantly reduced in iWAT of TG mice (Fig. 4a), while eWAT of TG mice displayed enhanced expression of peroxisome proliferator-activated receptor-γ2 (*Pparg2*) and reduced expression of carnitine palmitoyltransferase 1A (*Cpt1a*), involved in fatty acid oxidation (Fig. 4b). Expression of genes involved in glucose transport including glucose transporter 4 (*Slc2a4*), the early marker of differentiation Krüppel-like factor 5 (*Klf5*) and preadipocyte factor 1 (*Pref1*) was unchanged in both fat tissues (Fig. 4). Altogether, this suggests that late stages of differentiation might be affected by COX-2 overexpression in mature adipocytes of iWAT. In summary, this indicates depot-specific differences in response to COX-2 overexpression on adipocyte differentiation and maturation that might, at least in part, contribute to the different morphological and molecular phenotypes of iWAT and eWAT in TG mice.

### Overexpression of COX-2 in mature adipocyte modestly affects insulin-stimulated glucose disposal

**Figure 5:**
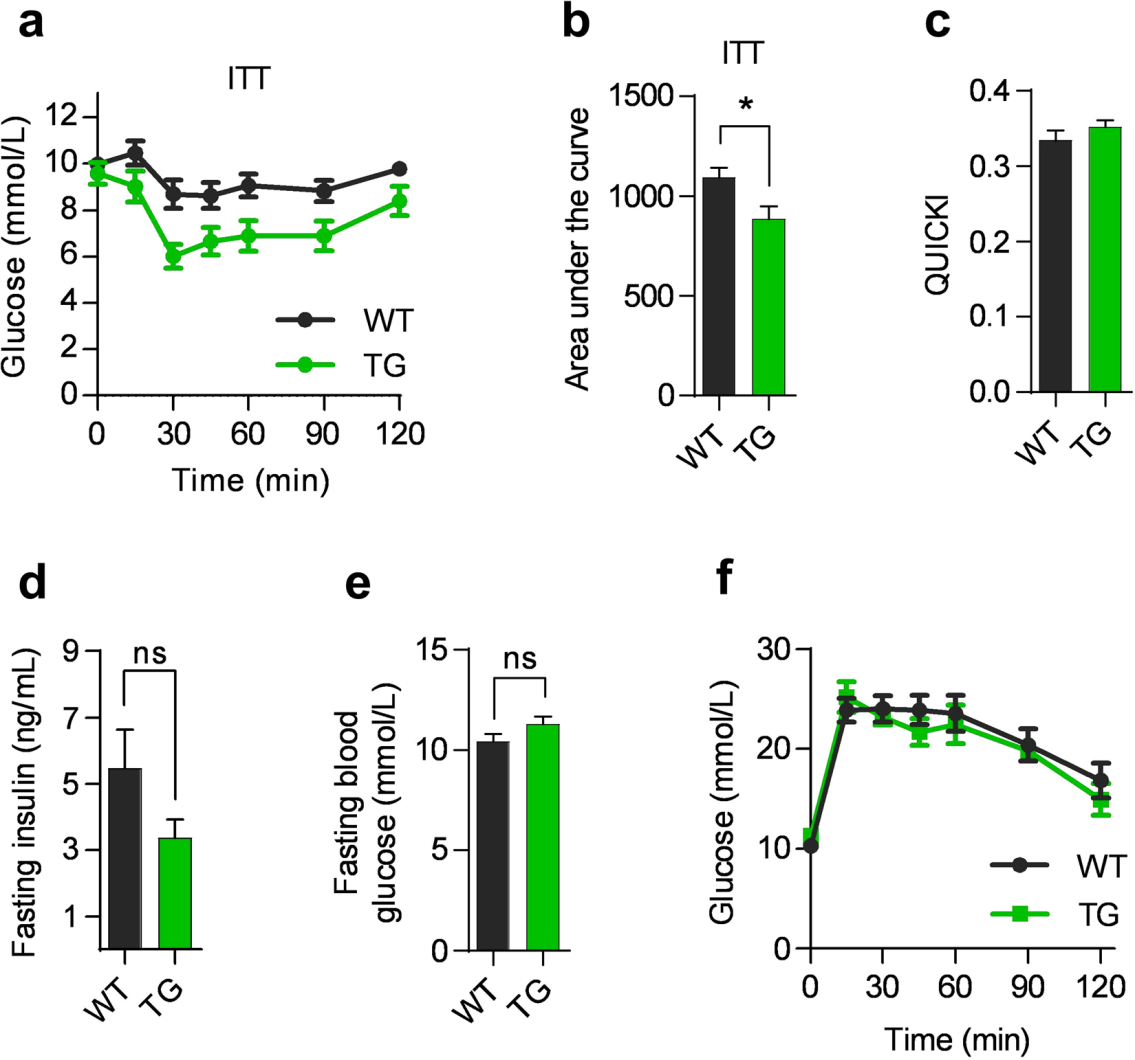
Overexpression of COX-2 in mature adipocyte modestly affects insulin-stimulated glucose disposal. (**a**) Insulin tolerance test (ITT) after 16 weeks on the HFD. (**b**) Area under the curve for ITT. (**c**) QUICKI at 15 weeks on the HFD. (**d**) Fasting insulin in ng/mL at GTT at week 15 on the HFD. (**e**) Fasting blood glucose after a 5 hour fast before GTT. (**f**) Glucose tolerance test (GTT) after 15 weeks on the HFD. All data from experiment 1. Students t-test, ns represents nonsignificant and *P ≤ 0.05. Mean ± SEM.

Small adipocytes are known to be metabolically beneficial, and since overexpression of COX-2 reduced the size of mature adipocytes in iWAT, we investigated whether this affected systemic glucose metabolism by performing glucose tolerance test (GTT) and insulin tolerance test (ITT). Overexpression of COX-2 was accompanied by improved insulin-stimulated glucose disposal as determined by ITT (Fig. 5a,b), but no improvement as determined by the quantitative insulin sensitivity check index (QUICKI) or the homeostatic model assessment (HOMA-IR) was observed (Figs 5C, S4a). Moreover, TG mice exhibited no change in fasting insulin (Fig. 5d), in fasting plasma glucose (Fig. 5e) or in glucose tolerance compared to WT mice (Fig. 5f). Finally, hepatic expression of glucose-6-phosphatase (*G6pc*) and phosphoenolpyruvate carboxykinase (*Pck1*) did not differ between TG and WT mice (Fig. S4b), suggesting similar regulation of hepatic gluconeogenesis in the mice. Taken together these observations indicated very minor changes in glucose homeostasis by overexpression of COX-2 in adipose tissues.

### Hepatic fat content and hepatocellular ballooning are decreased by COX-2 overexpression in adipose tissue

**Figure 6:**
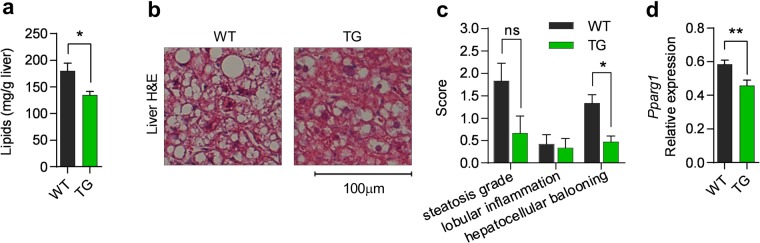
Hepatic fat content and hepatocellular ballooning are decreased by COX-2 overexpression in adipose tissue. (**a**) Liver lipid in mg/g. (**b**) Representative H&E stains of liver. (**c**) Hepatic steatosis grade, lobular inflammation and hepatocellular ballooning as evaluated by two independent pathologists. (**d**) Liver *Pparg1* mRNA expression. All data after 18 weeks of HFD (experiment 1). Students t-test, ns represents nonsignificant, *P ≤ 0.05 and **P ≤ 0.01. Mean ± SEM.

Since TG mice displayed reduced expansion of iWAT during HFD, we next investigated if COX-2 overexpression was associated with increased lipid storage and inflammation in the liver. Although liver mass was similar between the two genotypes (Fig. S5a), total lipid content was lower in TG mice than in WT mice (Fig. 6a,b). The content of triglycerides explained most of the difference in total hepatic lipid content, and no changes were found in other major lipid classes (Fig. S5b,c). Liver morphology reflected differences in lipid content with significant reduction in hepatocellular ballooning in TG mice (Fig. 6b,c), altogether demonstrating that decreased storage of triglycerides in iWAT did not result in increased storage of lipids in the liver. Further, hepatic *Pparg1* expression was significantly lower in TG mice (Fig. 6d), whereas no differences in expression of acyl-Coenzyme A dehydrogenase (*Acadm*), peroxisomal acyl-coenzyme A oxidase 1 (*Acox1*) or *Cpt1a* were found (Fig. S5D). Despite higher lipid content in WT mice, liver morphology indicated low lobular inflammation (Fig. 6c) supported by similar expression of *Ccl2* and *Tnfa* expression in the liver of TG and WT mice (Fig. 5Se). Collectively, we conclude that overexpression of COX-2 in adipose tissue reduced markers involved in hepatic steatosis in mice on HFD, whereas it had no effect on major liver inflammation parameters.

### TG mice exhibit increased number of eosinophils in eWAT and increased expression of type 2 immune markers in iWAT

**Figure 7:**
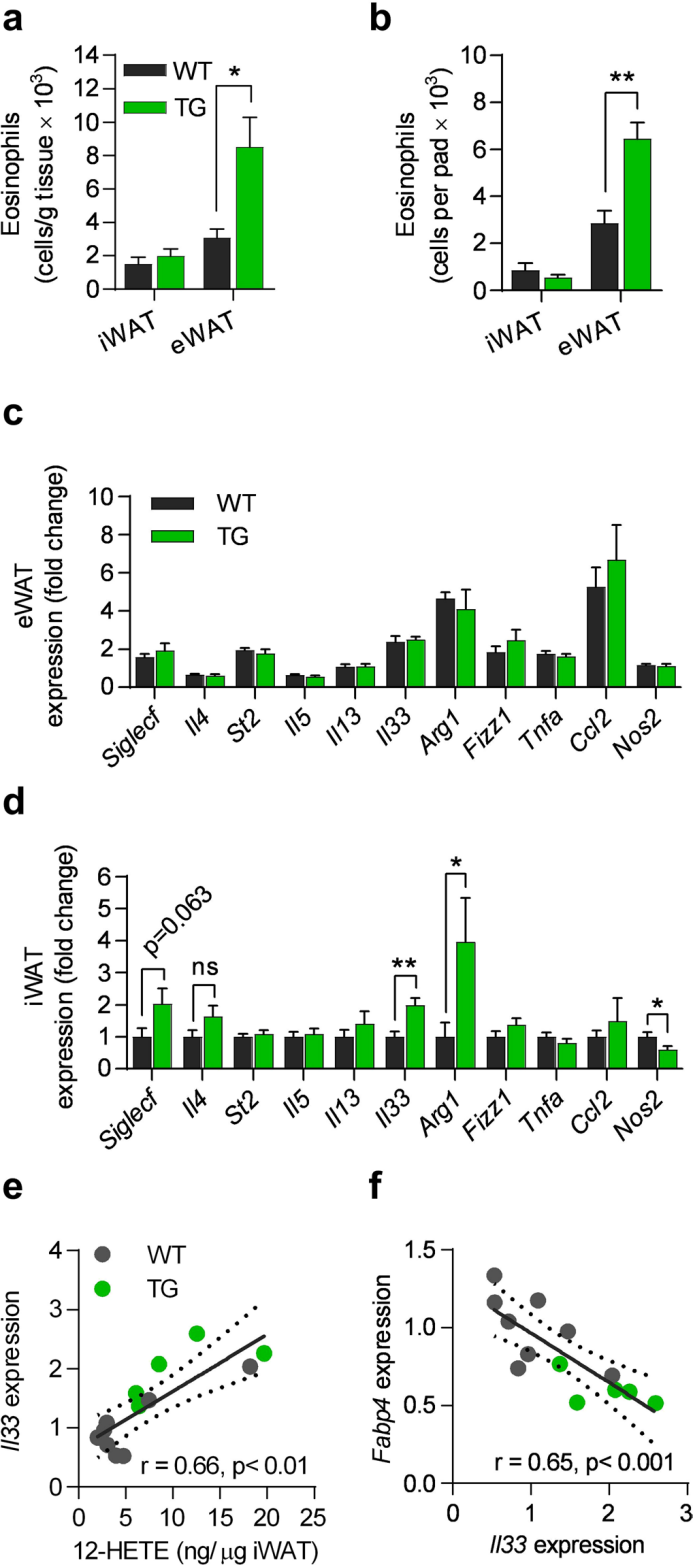
TG mice exhibit increased number of eosinophils in eWAT and increased expression of type 2 immune markers in iWAT. (**a**) Number of eosinophils per gram and (**b**) per pad of iWAT and eWAT. (**c**) S*iglecf*, *Il4*, *St2*, *Il5*, *Il13*, *Il33*, *Arg1*, *Fizz1*, *Tnfa*, *Ccl2*, and *Nos2* mRNA in eWAT, and (**d**) iWAT. (**e**) Spearman’s Rank correlation between 12-HETE and *Il33* expression, and (**f**) between *Il33* and *Fabp4* expression. All data after 18 weeks of HFD (experiment 1). Students t-test, ns represents nonsignificant, *P ≤ 0.05 and **P ≤ 0.01. Mean ± SEM.

Since many eicosanoids are potent chemoattractants and activators of especially type 2 immune cells^28–30^ that regulate adipose tissue and whole body metabolism^10,12,13^, we also profiled iWAT and eWAT to determine the abundances of macrophages, eosinophils and type 2 innate lymphoid cells (ILC2s) (Fig. S6 for gating strategy) as well as expression of key markers of these cell types (Fig. 7). In eWAT, but not in iWAT, overexpression of COX-2 resulted in 2-fold increase in the number of eosinophils (Figs 7a,b, S7g), whereas no differences in abundance of CD45^+^ cells, macrophages and type 2 innate lymphoid cells (ILC2) in iWAT or eWAT were found comparing TG and WT mice (Fig. S7a–f).

Next, we examined if changes in lipid metabolism and cell abundances were accompanied by differences in adipose tissue expression of key inflammatory indicators. Despite differences in eosinophil numbers in eWAT, expression of cytokines and markers associated with ILC2s, including ST2 (*St2*), IL-5 (*Il5*) and IL-13 (*Il13*), and eosinophils, including siglec-F (*Siglecf*) and IL-4 (*Il4*) was unaffected by adipocyte-selective overexpression of COX-2 (Fig. 7c). Neither were markers of macrophage polarization including the M2/alternatively activated macrophage markers IL-33 (*Il33*), *Arg1*, resistin-like beta (*Fizz1*), the M1 marker *Tnfa*, the chemoattractant *Ccl2*, or the type 1 marker inducible nitric oxide synthase (*Nos2*) affected by overexpression of COX-2 in eWAT (Fig. 7c). In contrast, we found increased expression of the alternatively activated macrophage markers *Il33* and *Arg1*, as well as lower expression of the type 1 marker *Nos2* in iWAT of TG mice (Fig. 7d), while no changes were seen for the other markers. IL-4 is mainly produced by eosinophils in WAT^12^, and consistent with this, *Il4* expression correlated positively with *Siglecf* (Fig. S7h), indicating increased eosinophil activation by COX-2-derived eicosanoids despite similar numbers of eosinophils in TG and WT mice (Fig. 7a,b). As IL-33 activates adipose tissue ILC2s, eosinophils, and consequently alternatively activated macrophages^13,31–33^, increased expression of IL-33 in iWAT indicated that one or more of these cell types might be activated within iWAT. Accordingly, we found increased expression of *Arg1*, known to be expressed in ILC2s and alternatively activated macrophages, whereas expression of cytokines produced by activated ILC2s including *Il13* and interleukin-5 (*Il5*) was unchanged in TG mice (Fig. 7d). In addition, the expression of *Il33* correlated with *Ptgs2* expression in WT mice (Fig. S7i). Interestingly, when examining the co-correlation structure between adipogenic genes, lipid mediators and immune markers in iWAT, we observed that concentrations of 12-HETE correlated with expression of *Il33* (Fig. 7e), in turn correlating inversely with *Fabp4* expression (Fig. 7f). This iWAT-interrelated structure could indicate a link from activation of IL-33, perhaps by 12-HETE, resulting in a reduction of the late stages of adipogenesis in iWAT. Taken together, COX-2 overexpression in mature adipocytes resulted in differential changes in iWAT and eWAT, coinciding with reduced markers of hepatic steatosis.

## Discussion

COX-2 activity has previously been shown to be required and sufficient for induction of UCP1 and browning in WAT^1,2^. However, we observed that COX-2 overexpression in mature adipocytes did not result in increased expression of *Ucp1* and browning in our model, despite markedly increased levels of several COX-2-generated lipid mediators, particularly in iWAT. Moreover, we found no increased respiration in TG mice at thermoneutral conditions, suggesting that enhanced expression and activity of COX-2 in mature adipocytes were insufficient for induction of *Ucp1* expression and uncoupled respiration. In previous experiments analysing the link between COX expression and browning, mice were housed at ambient temperature, not under thermoneutral conditions, indicating that COX activity in the absence of a beta-adrenergic tone might be insufficient to induce *Ucp1* expression. However, even when we exposed the TG mice to cold eliciting a β-adrenergic response, we observed no difference in *Ucp1* induction comparing WT and TG mice. Another reason for the lack of *Ucp1* induction could relate to the absence of a concomitant induction of COX-1 expression as we have shown that increased expression of both COX-1 and COX-2 is required to boost production of PGE_2_ and induction of UCP1 *in vitro*^1,15^. To what extent browning requires *de novo* recruitment of brown-like adipocytes in formally WAT termed beige or BRITE cells or can proceed via transdifferentiation of existing white adipocytes has been a matter of dispute^34^. Accumulated evidence would suggest that both processes may contribute, and therefore, our results suggest that enhanced COX-2 expression is not sufficient for either process to be initiated. Last and maybe most importantly remains the difference between mouse strains in relation to the role of COX-2 and COX-1 activity in browning. Much of the previous literature has been based on sv129 mice or transgenic mice on a mixed background. Here, our data based on inhibition of COX activity clearly point to a marked difference between the obesity resistant Sv129 mouse strain and the obesity prone C57BL/6 strain^1,8,9^.

Since the reported effect of COX-2 in relation to thermogenesis is likely mediated via the generation of lipid mediators, differences between studies might also be explained by differences in eicosanoid profiles. Vegiopoulos *et al*. found that *in vitro* expression of *Ucp1* in WAT mesenchymal progenitors was increased with the addition of a stable analogue of PGI_2_^2^. In our model, we did not observe increased levels of the stable PGI_2_ degradation product, 6-keto-PGF_1α_ in iWAT, which may, at least in part, explain the lack of *Ucp1* induction. Further, PGE_2_ has been suggested by us^1^ and others^35^ to induce *Ucp1* expression in WAT. Thus, subcutaneous administration of the Prostaglandin E receptor 4 (EP4) receptor agonist 16,16dmPGE_2_ increased *Ucp1* expression in iWAT^1^. Interestingly, despite at tenfold increase in PGE_2_ in iWAT, we did not observe increased *Ucp1* expression or browning in this model of adipocyte-selective overexpression of COX-2. Thus, it appears unlikely that increased energy expenditure contributed significantly to improvement in insulin sensitivity. Yet, as discussed above the lack of a concurrent increase in expression of *Ptgs1* may explain the lack of *Ucp1* induction and increased respiration in our model with adipocyte selective overexpression of COX-2.

Adipocyte-specific overexpression of COX-2 decreased iWAT mass and adipocyte size in TG mice compared to WT mice accompanied by lower expression of genes involved in terminal differentiation and maturation of adipocytes. It is well known that prostaglandins can modulate adipocyte differentiation via sustained COX-1 and COX-2 expression^36^. Thus, PGE_2_ and PGF_2α_ that were both increased in iWAT of the TG mice have been reported to inhibit adipogenesis^15–17,37^. In this regard it has been shown that mice with either decreased levels of PGF_2α_ or knock-out of the EP3 receptor exhibit identical phenotypes including higher body weight gain, larger eWAT and iWAT depots associated with hypertrophy of adipocytes, and impaired insulin sensitivity, but unaffected glucose tolerance^38,39^. However, we found no direct correlations between PGE_2_ and PGF_2α_ levels in iWAT and whole body metabolism in our model, suggesting that additional factors might be required for mediating systemic effects. Still, COX-2 overexpression in adipocytes marginally increased insulin-stimulated glucose clearance and tended to reduce body weight gain in mice fed a HFD. Increased subcutaneous fat mass associated with smaller adipocytes has been reported to improve insulin sensitivity as measured by hyperinsulinemic-euglycemic clamps, but not by insulin tolerance tests^12,40,41^. Interestingly, compared to WT mice, TG mice had lower liver lipid content and reduced hepatic steatosis. Increased subcutaneous fat also inhibits hepatic triacylglycerol accumulation and insulin suppression of hepatic glucose production^42,43^. Thus, although we observed smaller adipocyte size it seems unlikely that lower iWAT mass contributed to improvements in response to insulin injection. Rather, lower hepatic triacylglycerol level in COX-2 overexpressing mice may contribute to improvements in the insulin tolerance test. Thus, COX-2 mediated changes in adipocytes seemed to have off-target positive effects reducing storage of liver lipids and hepatic inflammation perhaps contributing to improved hepatic response to insulin. Given the effect of prostanoids on lipolysis in cultured adipocytes^24,27^ it is important to mention that this could also explain the lower adipocyte size in iWAT. However, hepatic triglyceride levels were decreased in COX-2 overexpressing mice indicating that possible effects of lipolysis did not redistribute fatty acids from adipose depots to the liver. Further, although the total fat mass was not significantly lower in COX-2 overexpressing mice compared to wildtypes, the average level indicated that other adipose depots apart from iWAT could have had a lower mass after HFD.

Recent insight into the pivotal roles of the immune system as a regulator of adipocyte function, whole body metabolism, and adaptive thermogenesis has highlighted the need for identifying processes and mediators affecting resident or recruited immune cells in adipose tissues^44^. COX-derived lipid mediators may play such a role, but due to the complexity and short lived nature, they are difficult to study. In response to forced expression of COX-2 and altered level of COX-generated eicosanoid in iWAT and eWAT, we observed differences in recruitment and activation of a number of immune cells. PGE_2_ inhibits eosinophil trafficking^45^, which at least in part may explain the difference between recruitment of eosinophils in iWAT and eWAT of TG mice, with higher levels of eosinophils and PGE_2_ in eWAT than in iWAT. Another possible cause of the observed COX-2-dependent differences in eosinophil abundance between WAT depots could relate to the ratios of PGE_2_ to 5-oxo-ETE, the latter being reported to act as a chemoattractant for human eosinophils^46^. Here we found that 5-oxo-ETE was increased in eWAT, but not in iWAT, coinciding with increased levels of eosinophils in eWAT, but not in iWAT. By contrast we observed a higher activation of eosinophils in iWAT as measured by *Il4* expression. Interestingly, both PGF_2α_ and PGD_2_ are agonists for the prostaglandin DP2 (CRTH2) receptor^47^, expressed by eosinophils and ILC2s. We found 3.7-fold higher levels of PGF_2α_ in iWAT than in eWAT in TG mice; a factor that might be important for the observed difference between iWAT and eWAT in activity of especially eosinophils.

Other lipid mediators that might be important for the observed differences between fat depots are 12-HEPE and 12-HETE that were specifically increased in iWAT of TG mice. 12-HETE was found to positively correlate with *Il33* in iWAT, which, in turn correlated negatively with *Fabp4* expression levels. These interlinked factors could indicate a role for 12-HETE in inducing expression of *Il33*. To our knowledge, a possible effect of 12-HETE on *Il33* expression levels has not been reported. The role of IL-33 in reducing adipocyte differentiation has previously been shown by others^48–50^. Other recent studies point to a role for IL-33 in regulation of adipose tissue homeostasis and browning via ILC2-mediated processes^10,11,31,50^. Despite increased *Il33* expression, we found no increase in ILC2 activity or number and no browning. Expression of *Il33* and ST2 has also been reported to be increased in adipose tissue of obese mice and humans^10^, while the number of ST2-expressing cell has been reported to be reduced in WAT in obese animal models^11,31^, illustrating possible yet unresolved issues regarding IL-33 and its influence on WAT metabolism in homeostatic conditions and during obesity development.

Interestingly and contrary to the general notion that COX-2 in adipose tissue enhances pro-inflammatory processes in WAT, our results indicated that COX-2 activity in eWAT increased the number of cells associated with anti-inflammatory effects. Additionally, we identified reduction in the type 1 mediator iNOS and induction of anti-inflammatory type 2 based immune mediators in iWAT including IL-4, which is typical for eosinophils, and Arg-1, which is expressed in alternatively activated macrophages. IL-4 and 13-HODE are both reported to increase PPARγ activity in macrophages, which is associated with alternative activation, limiting inflammation, and insulin sensitivity^51,52^. Overall, this implies that WAT-dependent COX-2 activity might play a role in overall body metabolism, although we did not identify such correlations in the present study. The results on eosinophil accumulation and differences in depot specific activity warrant further investigation and may contribute to explain reported disparate effects of eosinophils in adipose tissue on glucose metabolism in HFD fed mice^12,41^.

We did not explore mechanisms behind the different effects of COX-2 overexpression in relation to eicosanoid synthesis in the two WAT depots. In light of the positive effects on immune regulation this should be further explored in the future. Since dietary fat composition regulates the cellular phospholipid profile^53^ which in turn affects the pattern of prostanoid synthesis by cyclooxygenases^53^, the dietary fat source may have contributed to our eicosanoid phenotype. Finally, depot specific activity of phospholipase A2 might also explain differences between eWAT and iWAT.

In summary, we conclude that COX-2 activity in WAT improves the inflammatory profile of subcutaneous adipose tissue, but does not increase expression of UCP1 associated with uncoupled respiration and browning. Our results do not exclude that induction of COX-2 expression and enhanced cyclooxygenase activity are involved in browning, but show that enhanced COX activity and increased levels of prostaglandins in mature adipocytes are insufficient to promote transdifferentiation of existing adipocytes at thermoneutrality^34^. It still remains a possibility that COX expression is required for recruitment and differentiation of beige/BRITE adipocytes^2^ or that COX activity in infiltrating immune cells plays a role. Importantly, our finding that selective overexpression of COX-2 in mature adipocytes elicited positive off-target effects on hepatic steatosis indicates the presence of a complex crosstalk between adipocytes and hepatocytes possibly orchestrated by adipocyte-derived prostaglandins in synergy with additional cues that remain to be identified.

## Methods

### Animals

We created a construct carrying the coding region of the murine *Ptgs2* gene under the control of a truncated adiponectin promotor^26^. This was inserted in mice on a C57BL/6NTac background, resulting in the Tg(Ptgs2)Wfrm strain. Two founders were generated (261 and 263). All data described here were generated using the 263 strain, which expressed the highest level of COX-2.

Four independent experiments were conducted: In the first, male transgenic and wild type (unless otherwise indicated: TG, n = 5; WT, n = 9) littermates were co-housed at room temperature with a 12-hour light/dark cycle with free access to chow and water. At 8 weeks of age, mice were moved to thermoneutrality (28–30 °C) and after 2 weeks of adaptation changed to a HFD (60%E fat, research diets D12492, Ssniff, Germany) for 17 weeks followed by three days of adaptation and 4 days of measurement in metabolic chambers before dissection. Food intake was recorded twice a week and mice were weighed once a week. The second experiment (TG and WT, n = 10) was a repetition of the first, except that GTT, ITT and dissection was performed after one, two and three weeks on HFD. The third experiment (TG and WT, n = 7) was a repetition of the first, except that mice were single-caged and placed at 16 °C at 13 weeks of age and terminated after one week. Data from the third experiment were used for determination of cold induced *Ucp1* expression. The fourth experiment (TG, n = 7–8; WT, n = 7) was a repetition of the first, except that mice were single-caged and placed in metabolic chambers after 4 weeks of HFD. Data from the fourth experiment were used for determination of energy intake, energy expenditure upon cold exposure, and fat digestibility.

At dissection, mice were anesthetized using isoflurane (Isoba-vet, Schering-Plough, UK) in the fed state and euthanized by cardiac puncture. Tissues for flow cytometry were processed immediately and tissues for histology were fixed in 4% paraformaldehyde in phosphate buffer; all other tissues were immediately frozen in liquid nitrogen, and stored at −80 °C. All animal breeding and experimentation was approved by the Danish Animal Experiment Inspectorate (Ref. No.: 2012-15-2935-00028 and 2014-15-2934-01027) and in accordance with EU Directive 2010/63/EU for animal experiments. Glucose and insulin tolerance test. For GTT, mice were fasted for 5 hours and injected i.p. with 3 g glucose/kg lean mass. For ITT, mice were fasted for 2 hours and injected i.p. with 1.0 U human insulin (Actapid, Novo Nordisk, Denmark)/kg lean mass. For all tests, blood was collected from the tail vein of conscious animals and blood glucose was measured using a glucometer (Contour XT, Bayer, USA) at baseline and at the indicated time points.

### Indirect calorimetry

After a 2 day acclimatization period, O_2_ and CO_2_ gas exchange measurements were obtained for a 4 day period from each mouse using the open circuit chambers Labmaster system (TSE Systems, Germany).

### Fat oxylipin quantification

Samples were lyophilized and resuspended in 1 ml 10% methanol containing deuterated internal standards (Standards are listed in Suppl. Table 1) followed by an extraction using solid reverse phase extraction columns (Bond Elut Plexa, Agilent). Fatty acid derivatives were eluted into 1.0 ml of methanol, lyophilized and resuspended in 100 µl of water/acetonitrile/formic acid (70:30:0.02, v/v/v; solvent A) and analysed by LC-MS/MS on an Agilent 1290 separation system. Samples were separated on a Synergi Hydro reverse-phase C18 column (2.1 × 250 mm; Phenomenex) using a gradient as follows: flow rate 0.3 µl/min, 1 min (0% solvent B: acetonitrile/isopropyl alcohol, 50:50, v/v;), 3 min (25% solvent B), 11 min (45% solvent B), 13 min (60% solvent B), 18 min (75% solvent B), 18.5 min (90% solvent B), 20 min (90% solvent B), 21 min (0% solvent B). The separation system was coupled to an electrospray interface of a QTrap 5500 mass spectrometer (AB Sciex). Compounds were detected in scheduled multiple reaction monitoring mode. For quantification a 12-point calibration curve for each analyte was used. Data analysis was performed using Analyst (v1.6.1) and MultiQuant (v2.1.1) (AB Sciex, Switzerland). Oxylipins measured are detailed in Suppl. Tables 2–3.

### Histology

Fixed samples and paraffin embedded sections of liver, eWAT, and iWAT were stained with hematoxylin and eosin. Adipocyte size was determined in hematoxylin/eosin-stained sections as the mean cell area (in μm^2^) of 200 randomly selected adipocytes on digital images acquired at ×10 by a Nikon Eclipse e800 light microscope (Nikon, Tokyo, Japan) using a digital image system (LUCIA Imaging, v 4.82, Czech Republic). Evaluation of NASH grading score of steatosis, inflammation and hepatocellular ballooning in liver histology sections was done by a blinded, independent laboratory using 3 liver sections per sample.

### Quantitative Reverse Transcriptase Polymerase Chain Reaction (qRT-PCR) and Western Blotting

Total RNA from iWAT, eWAT and liver was extracted using Trizol according to the product protocol (Invitrogen, USA). RNA concentration and purity were determined using the Nanodrop Spectrophotometer ND-2000. Reverse transcription was carried out on 0.5 µg of RNA from fat and 1 µg of RNA from liver using RevertAid Reverse Transcriptase cDNA Synthesis Kit (Thermo Fisher, USA), random hexamer primer, reaction buffer, and dNTP Mix in 20 µL according to product protocol and diluted 10–100 fold for further use. Quantitative RT-PCR was performed using SensiFAST SYBR Lo-ROX kit (Bioline, USA) on an Mx3000P qPCR system (Agilent Technologies, USA). Primer sequences are detailed in Suppl. Table 4. The following PCR thermal profile was used with annealing temperature depending on primer melting temperature: 95 °C for 5 min; 40 cycles of 95 °C for 15 sec, 55–63 °C for 20 sec, 72 °C for 15 sec followed by melting curve preparation: 95 °C for 1 min, 55–63 °C for 1 min and ramping to 95 °C. Expression was subsequently normalized to TATA-binding protein (*Tbp)*. Western blotting was performed as described earlier^54^ using antibodies against Vinculin (V9131, Sigma, USA), COX-2 (Ab15191, Abcam, United Kingdom) and UCP-1 (Ab3036, Abcam, United Kingdom).

### Tissue lipid extraction and lipid class analysis

Total lipid was extracted from liver samples with chloroform:methanol, 2:1 (v/v) and 0.01% butylated hydroxytoluene (BHT). After filtration, evaporation and addition of chloroform with 0.01% BHT samples were quantified on a High performance thin layer chromatography system (Camag, Switzerland) using standards for L-α-lysophosphatidylcholine, springomyelin, phosphatidylcholine, phosphatidylinositol, phosphatidylethanolamine, cholesterol, free fatty acid (linolenic acid), triacylglycerol (trilinolenin)(Sigma, USA) and phosphatidylserine, phosphatidic acid, and cardiolipin (Avanti, USA).

### Flow cytometry

Dissected adipose tissue was cut into small pieces and suspended in RPMI (Sigma, USA) with 20% heat-inactivated FCS (Lonza, Switzerland) before homogenization for 1 hour in a shaker-incubator at 37 °C with 2 mg/mL collagenase II (C6885, Sigma, USA). Homogenized adipose tissue was processed as previously described^55^ using FACS buffer (PBS with 1% heat-inactivated FCS and 0.1% NaN_3_) without fixation and permeabilisation. Cells were recorded on a LSRII (BD Biosciences, USA) flow cytometer, and data further analysed using Flowjo software (V10.0.7, Treestar). Gating strategies are shown in Fig. S6. The following antibodies for surface staining were used: CD45/PerCP (BioLegend, 30-F11), Siglec-F/PE (BD, E50-2440), CD11b/V500 (BD, M1/70), F4/80/APC (BioLegend, BM8), CD11c/APC-Cy7 (BD, HL3), CD45/AF647 (BD, 30-F11), CD4/AF488 (eBioscience, GK1.5), CD8a/FITC (BD, 53–6.7), CD11b/FITC (eBioscience, M1/70), CD49b/FITC (eBioscience, HMa2), F4/80/FITC (eBioscience, BM8), NK1.1/FITC (BD, PK136), FcεR1/PerCP-eF710 (eBioscience, MAR-1), CD19/PerCP-Cy5.5 (eBioscience, 1D3), CD11c/PE-cy7 (BD, HL3), ST2-biotin (MD biosciences, 101001B), Streptavidin-PE (eBioscience).

### Statistics

All results are shown as mean ± SEM unless otherwise indicated. Statistical analyses of physiological and gene expression data were performed with GraphPad Prism v6.07 (GraphPad Software, Inc., USA). Student’s two-tailed t-test was used to compare differences between genotypes. Statistical analysis of oxylipins was performed using Mann-Whitney U test adjusting for false-discovery rate in R-3.1.3 and outliers were screened using Grupps test. The Spearman Rank test was used for correlations as indicated. A significance level of P ≤ 0.05 was used for all tests. Unless otherwise stated, n = 5 for TG and 9 for WT. Statistical significances are denoted with stars; *P ≤ 0.05, **P ≤ 0.01, ***P ≤ 0.001.

## Supplementary information

Supplementary figures and tables
