# Supplementary material for: Overexpression of cyclooxygenase-2 in adipocytes reduces fat accumulation in inguinal white adipose tissue and hepatic steatosis in high-fat fed mice
Source: Sci Rep. 2019 Jun 20;9:8979. doi: 10.1038/s41598-019-45062-w (PMC6586826; doi:10.1038/s41598-019-45062-w)
Supplement: Supplementary file 1 — Supplementary figures and tables [file 41598_2019_45062_MOESM1_ESM.pdf]

SUPPLEMENTAL INFORMATION FOR

Overexpression of cyclooxygenase-2 in adipocytes reduces fat accumulation in inguinal white adipose tissue and hepatic steatosis in high-fat fed mice

Niels Banhos Danneskiold-Samsøe, Si Brask Sonne, Jeppe Madura Larsen, Ann Normann Hansen, Even Fjære, Marie Sophie Isidor, Sidsel Petersen, Jeanette Henningsen, Ilenia Severi, Loris Sartini, Yvonne Schober, Jacqueline Wolf, W. Andreas Nockher, Christian Wolfrum, Saverio Cinti, Christian Sina, Jacob Bo Hansen, Lise Madsen, Susanne Brix, Karsten Kristiansen

To whom correspondence should be addressed.
E-mail: nds@bio.ku.dk, lise.madsen@hi.no, sbp@bio.dtu.dk, kk@bio.ku.dk

This PDF file includes:

- Figures S1-S7
- Supplementary methods
- Supplementary table S1-S4

Figure S1.

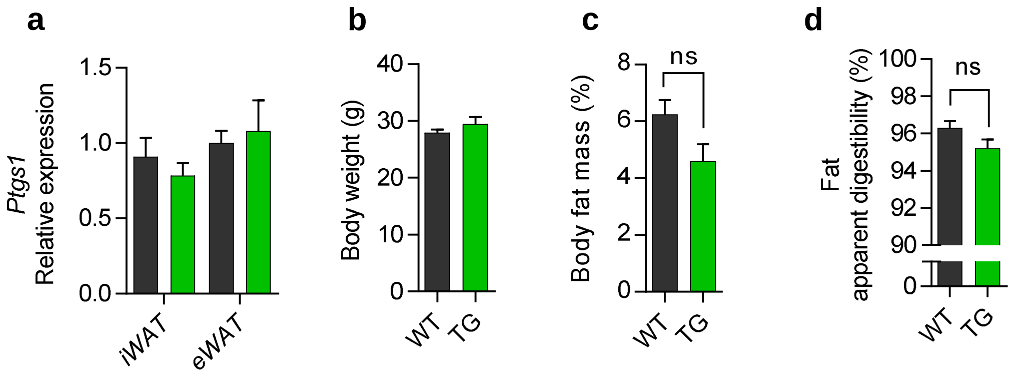

Figure S1, Overexpression of COX-2 in mature adipocytes leads to a marginally lower body weight gain in response to high fat feeding. a-c present results from experiment 1. **(a)** Adipose tissue *Ptgs1* mRNA expression in mice after 18 weeks on the HFD. **(b)** Body weight and **(c)** body fat mass of mice on fed the chow diet at 10 weeks of age after 2 weeks at 30°C before commencement of the HFD feeding. **(d)** Apparent digestibility of fat in feed after 2 weeks of HFD in single housed mice (n = 7)**.**

Figure S2.

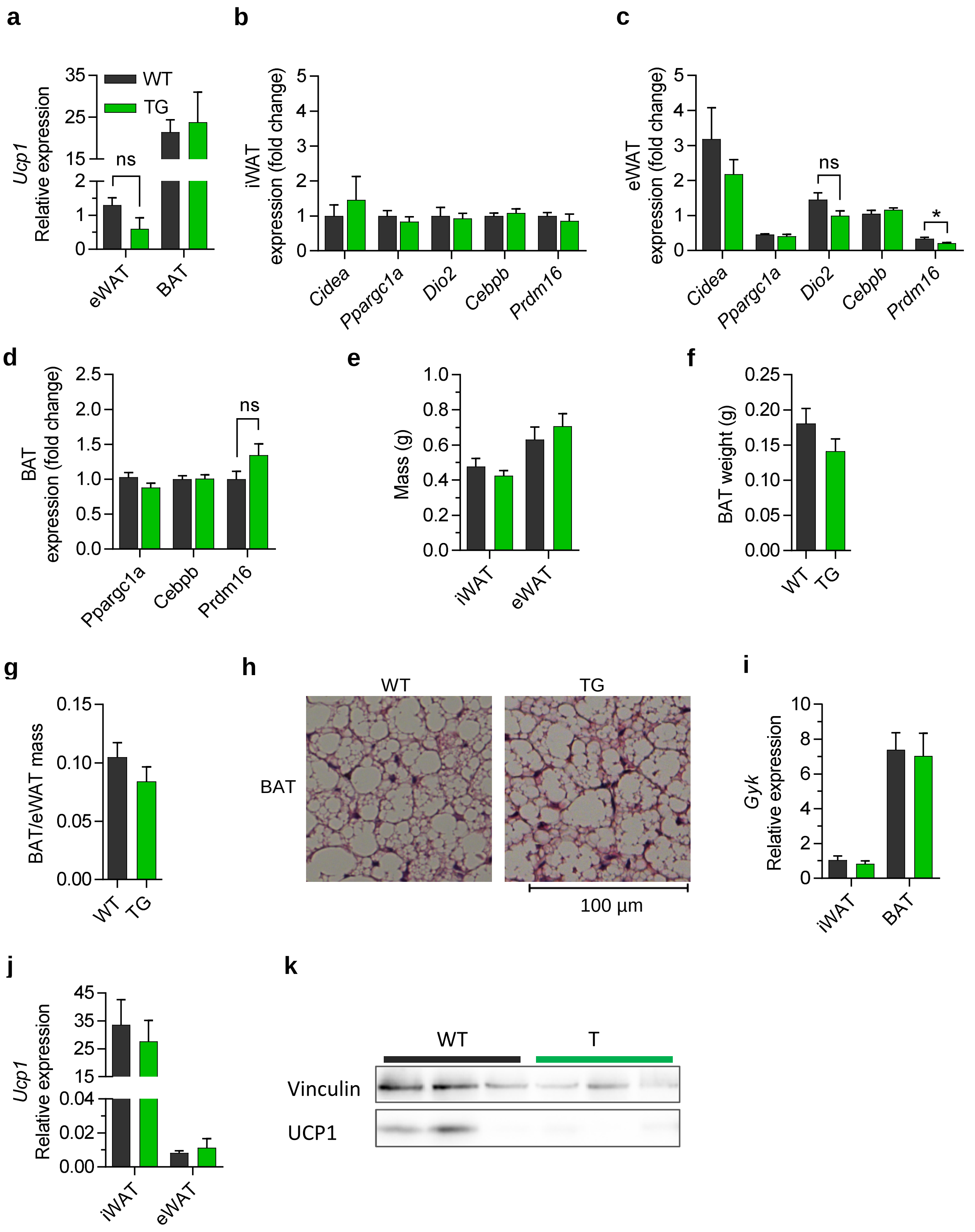

Legend on next page

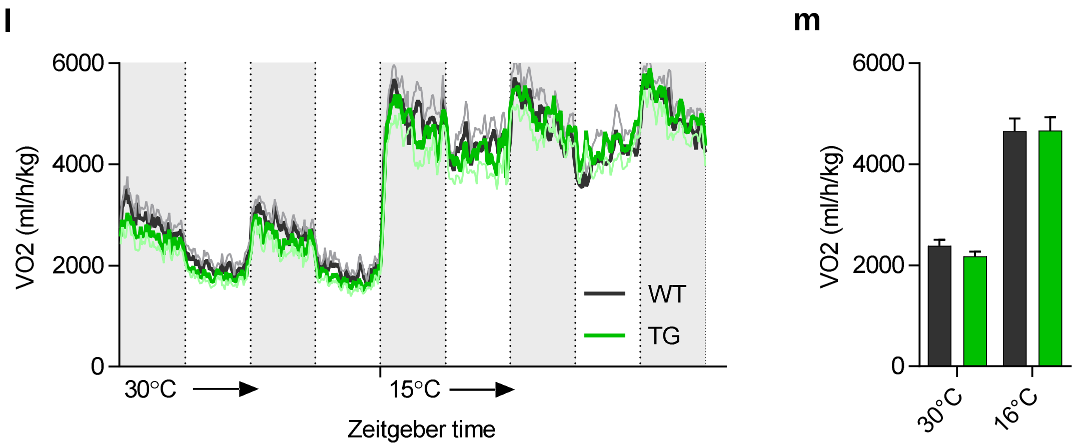

Figure S2. Overexpression of COX-2 in mature adipocytes does not induce expression of *Ucp1* but still reduces iWAT mass and alters adipocyte size. a-d and f-i present results from experiment 1 after 18 weeks on HFD. **(a)** *Ucp1* mRNA in eWAT and BAT (interscapular brown adipose tissue) in HFD mice at thermoneutrality**. (b)** *Cidea*, *Ppargl a*, *Dio2*, *Cebpb* and *Prdm16* mRNA in iWAT and (**c**) in eWAT**.** (**d**) *Ppargl a*, *Dio2*, *Cebpb* and *Prdm16* mRNA in BAT. **(e)** iWAT and eWAT mass after three weeks on HFD (experiment 2) (n=10). (**f**) Mass of BAT after 18 weeks on HFD (experiment 1)**. (g)** Relative mass of BAT compared to eWAT. **(h)** Representative H&E sections of BAT. **(i)** *Gyk* mRNA in iWAT and BAT. **(j)** *Ucp1* mRNA in iWAT and BAT (experiment 3) after one week of cold exposure in HFD mice (n=7)**. (k)** Western Blot of iWAT after one week of cold exposure (experiment 3). (l) Oxygen consumption rate (VO_2) in ml/h/kg at 30°C and 15°C in single caged (experiment 4) after 4 weeks on the HFD (n = 8). **(m)** Average VO_2 at 30°C and 15°C from l. Students t-test, ns represents nonsignificant. Mean ± SEM.

Figure S3.

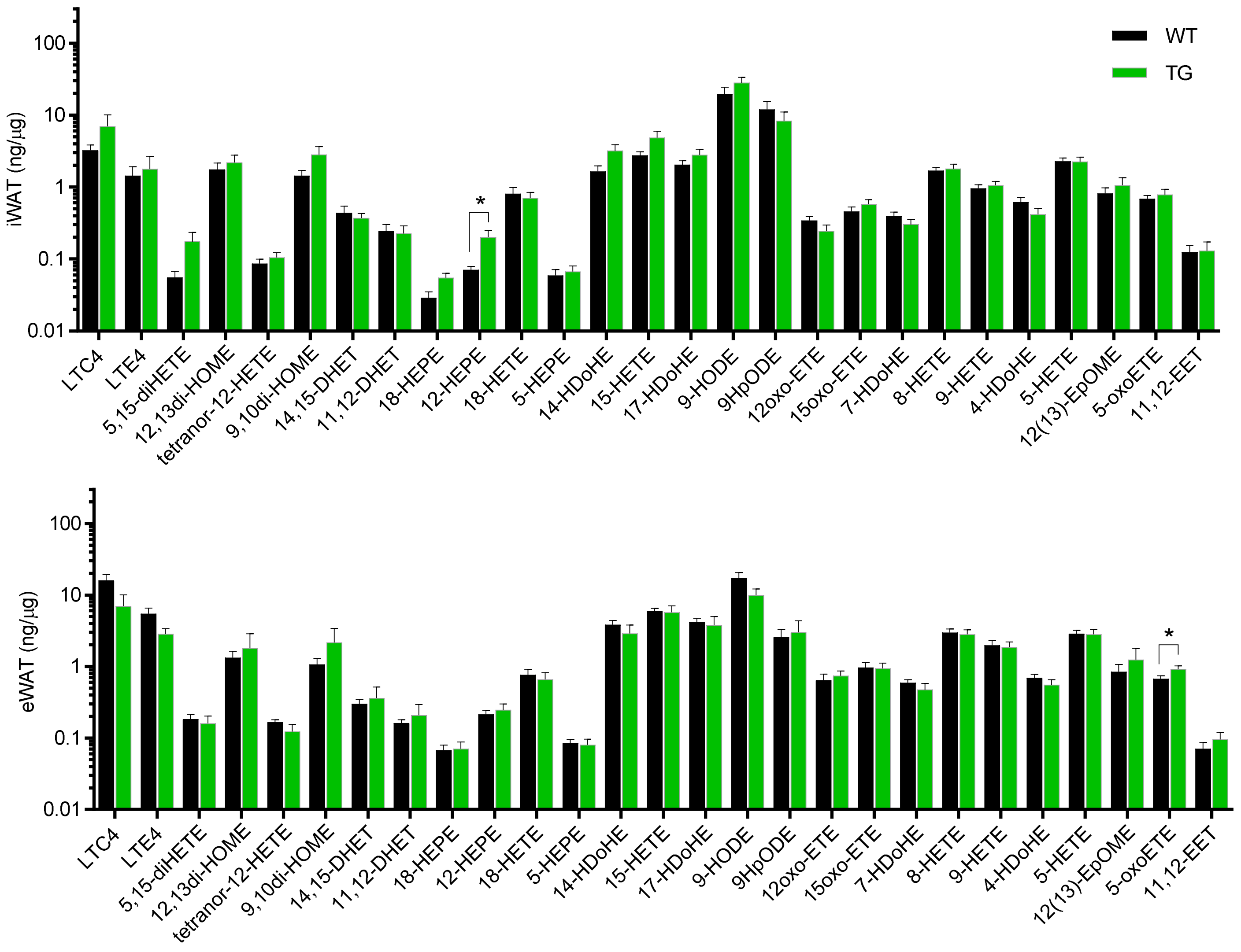

Figure S3. Eicosanoids produced by COX-2 in iWAT and eWAT are selectively increased in TG mice. Concentration of indicated eicosanoids in iWAT and eWAT after 18 weeks of HFD in mice (experiment 1). Mann-Whitney U test for differences between groups with adjustment for false-discovery rate. * represents P≤0.05.

Figure S4

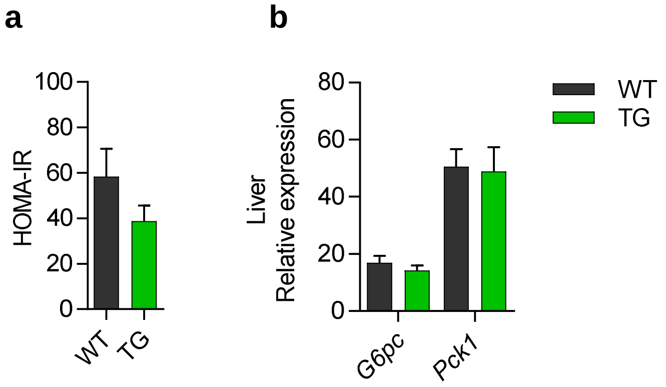

Figure S4. Overexpression of COX-2 in mature adipocytes modestly affects insulin-stimulated glucose disposal. (a) Homeostatic model assessment-insulin resistance (HOMA-IR) at time of GTT at week 15 on HFD. **(b)** Expression of *G6pc* and *Pck1* mRNA in liver. All data from experiment 1. Students t-test, ns represents nonsignificant. Mean ± SEM.

Figure S5

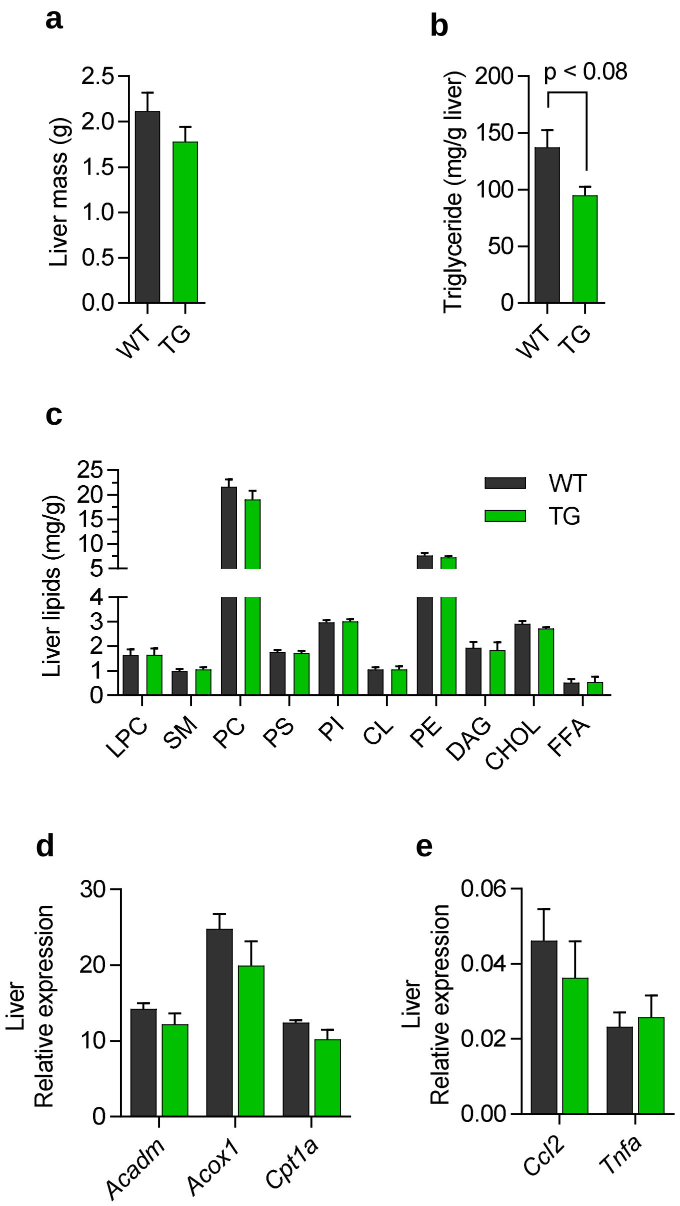

Figure S5. Hepatic fat content and hepatocellular ballooning were decreased by COX-2 overexpression in mature adipocytes. (a) Liver mass in gram. **(b)** Total triglyceride in mg/g liver. **(c)** Other liver lipids in mg/g liver LPC: Lysophosphatidylcholine, SM: Sphingomyelin, PC: Phosphatidylcholine, PS: Phosphatidylserine, PI: Phosphatidylinositol, CL: Cardiolipin, PE: Phosphatidylethanolamine, DAG: Diacylglycerol, CHOL: Cholesterol, FFA: Free fatty acid. **(d)** *Acadm*, *Acox1* and *Cpt1a* mRNA in liver. **(e)** *Ccl*2 and *Tnfa* mRNA in liver. All data from experiment 1. Students t-test. Mean ± SEM.

Figure S6

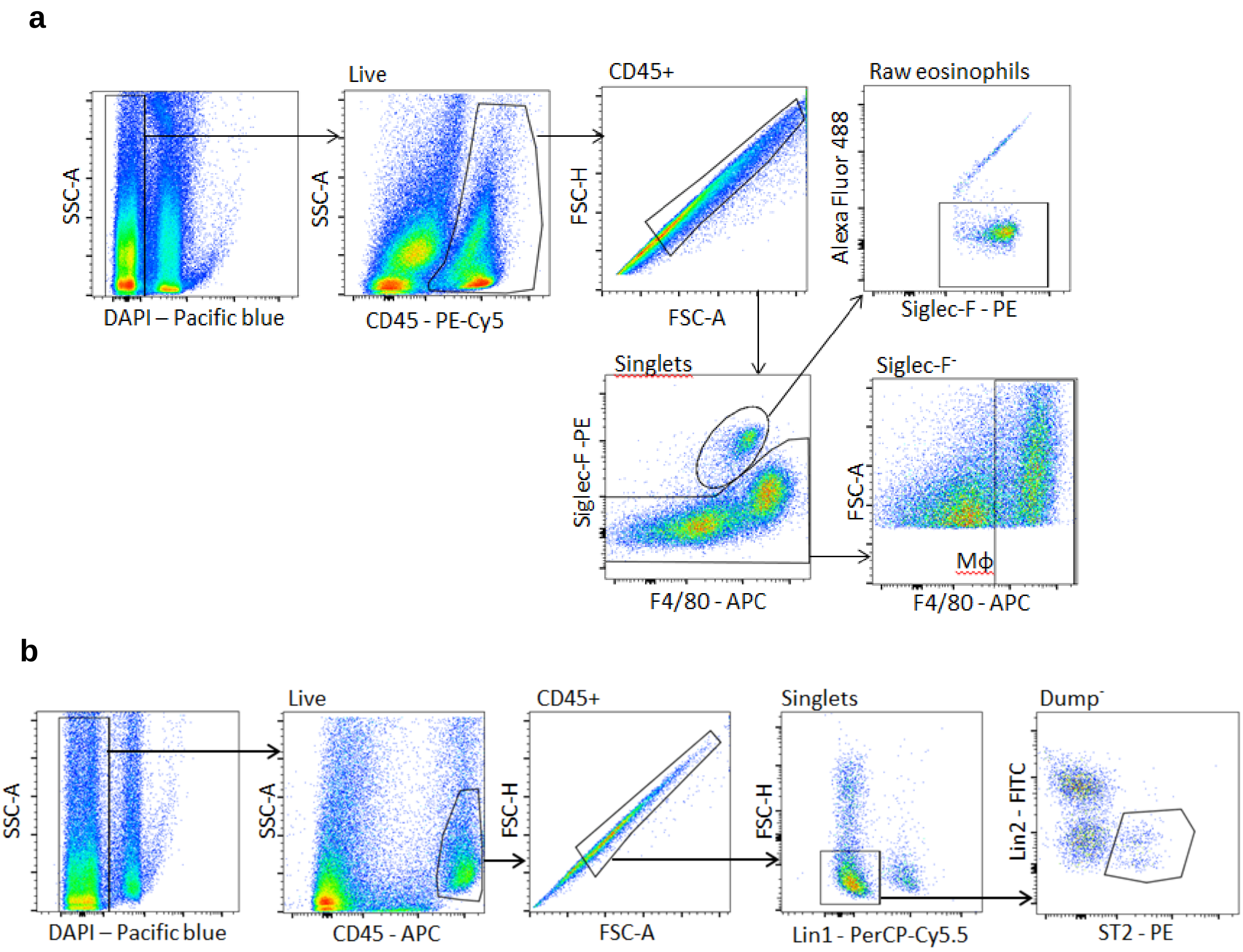

Figure S6. Gating strategy for immune cells in iWAT and eWAT. (a) Representative gating strategy for eosinophils and macrophages, and **(b)** for ILC2 cells. Lineage cocktail 1 (Lin1): FcεR1, CD19, lineage cocktail 2 (Lin2): CD4, CD8α, CD11b, CD49b, F4/80, NK1.1.

Figure S7

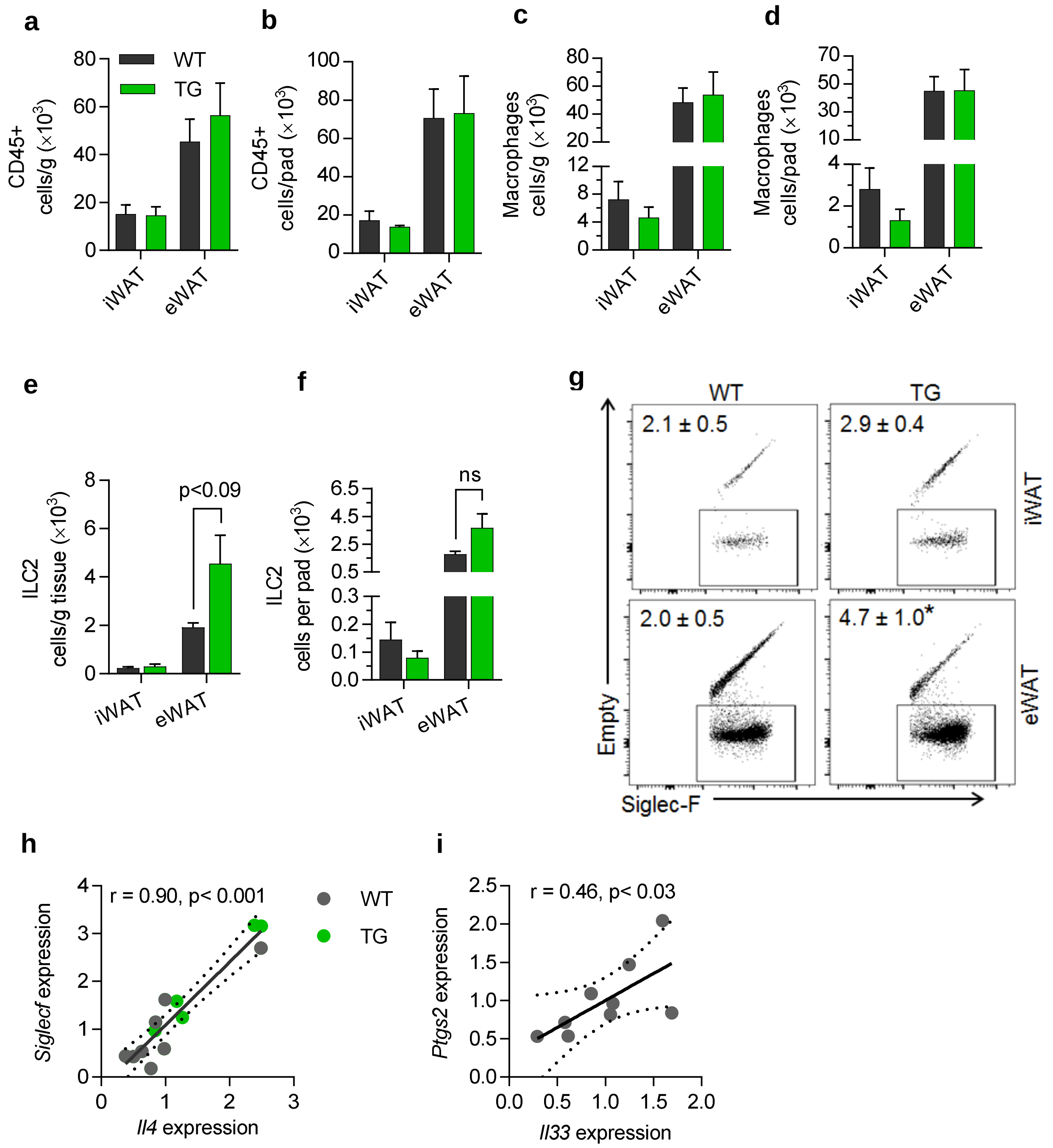

Figure S7. Increased number of eosinophils in eWAT and increased expression of type 2 immune markers in iWAT in TG mice. (a) $CD45^{+}$ cells per gram, and **(b)** per fat depot of iWAT and eWAT. **(c)** Macrophages per gram, and **(d)** per depot of iWAT and eWAT. **(e)** ILC2 cells per gram, and **(f)** per fat depot**. (g)** Abundance of eosinophils in per cent in SVF of iWAT and eWAT. **(h)** Correlation between *Il4* and *Siglecf* and **(i)** *Il33* and *Ptgs2* mRNA expression in iWAT. All data from experiment 1 after 18 weeks of HFD. Students t-test, ns represents nonsignificant. Mean ± SEM.

SUPPLEMENTAL PROCEDURES

Apparent digestibility of fat. Total lipid was extracted from feces and quantified in samples collected from a total of 5 days following 2 weeks of adaptation to HFD in a separate group of single-housed mice. Total feces was collected and frozen at -20°C until analysis. Samples were weighed and 7 ml n-heptane was added to 0.7 g of feces followed by homogenization, centrifugation, evaporation and registration of free fatty acids. HCl and n-heptan was added to the remaining solid phase and the samples were heated at 90°C for 2 hours. After cooling, samples were spun down and the organic phase was transferred to a new tube. Hydromatrix and sample slurry was added to a liquid-liquid extraction column. After 5 min of incubation lipids were eluted using petroleum ether in a Büchi syncore evaporation unit. Samples were subsequently heated to 103°C for 30 min and dried in a desiccator before being weighing remaining fat. Total fat was calculated by adding free fatty acids and remaining fat after desiccation.

Table S1. Lipid mediator standards used for quantification of lipid mediators.

Abbreviation	Full name/Synonym	Systematic name
6-keto-PGF1a-d4	6-keto-PGF1α-d4	6-oxo-9α,11α,15S-trihydroxy-prost-13E-en-1-oic-3,3,4,4-d4 acid
RVE1-d4	Resolvin E1-d4	5S,12R,18R-trihydroxy-6Z,8E,10E,14Z,16E-eicosapentaenoic-6,7,14,15-d4 acid
TXB2-d4	Thromboxane B2-d4	9α,11,15S-trihydroxy-thromba-5Z,13E-dien-1-oic-3,3,4,4-d4 acid
iPF2a-d11	5-iPF2α-VI-d11	(±)5,9α,11α-trihydroxy-(8β)-prosta-6E,14Z-dien-1-oic-16,16,17,17,18,18,19,19,20,20,20-d11 acid
PGF2a-d4	Prostaglandin F2a-d4	9α,11α,15S-trihydroxy-(8β)-prosta-5Z,13E-dien-1-oic-3,3,4,4-d4 acid
PGE-2-d4	Prostaglandin E2-d4	9-oxo-11α,15S-dihydroxy-prosta-5Z,13E-dien-1-oic-3,3,4,4-d4 acid
PGD2-d4	Prostaglandin D2-d4	9α,15S-dihydroxy-11-oxo-prosta-5Z,13E-dien-1-oic-3,3,4,4-d4 acid
13,14-diOH-15-keto-PGE2-d4	13,14-dihydro-15-keto Prostaglandin E2-d4	9,15-dioxo-11α-hydroxy-prost-5Z-en-1-oic-3,3,4,4-d4 acid
13,14-diOH-15-keto-PGF2a-d4	13,14-dihydro-15-keto Prostaglandin F2a-d4	9α,11α-dihydroxy-15-oxo-prost-5Z-en-1-oic-3,3,4,4-d_4 acid
13,14-diOH-15-keto-PGD2-d4	13,14-dihydro-15-keto Prostaglandin D2-d4	9α-hydroxy-11,15-dioxo-prost-5Z-en-1-oic-3,3,4,4-d_4 acid
LTC4-d5	Leukotriene C4-d5	5S-hydroxy-6R-(S-glutathionyl)-7E,9E,11Z,14Z-d5-eicosatetraenoic acid
LTE4-d5	Leukotriene E4-d5	5S-hydroxy-6R-(S-cysteinyl)-7E,9E,11Z,14Z-eicosatetraenoic-19,19,20,20,20-d5 acid
PGB2-d4	Prostaglandin B2-d4	9-oxo-15S-hydroxy-prosta-5Z,8(12),13E-trien-1-oic-3,3,4,4-d4 acid
LTB4-d4	Leukotriene B4-d4	5S,12R-dihydroxy-6Z,8E,10E,14Z-eicosatetraenoic-6,7,14,15-d4 acid
12,13-di-HOME-d4	(±)12(13)-DiHOME-d4	(±)12,13-dihydroxy-9Z-octadecenoic-9,10,12,13-d4 acid
9,10-di-HOME-d4	9,10-diHOME-(d4)	9,10-dihydroxy-12Z-octadecenoic acid-(d4)
15-deoxi-12,14-PGJ2-d4	15-deoxy-Δ12,14-PGJ2-d4	11-oxo-prosta-5Z,9,12E,14E-tetraen-1-oic-3,3,4,4-d4 acid
20-HETE-d6	20-hydroxy Arachidonic Acid-d6	20-hydroxy-5Z,8Z,11Z,14Z-eicosatetraenoic-16,16,17,17,18,18-d6 acid
13-HODE-d4	13(S)-HODE-d4	13S-hydroxy-9Z,11E-octadecadienoic-9,10,12,13-d4 acid
15-HETE-d8	15(S)-HETE-d8	15(S)-hydroxy-5Z,8Z,11Z,13E-eicosatetraenoic-5,6,8,9,11,12,14,15-d8 acid
9-HODE-d4	9(S)-HODE-d4	9S-hydroxy-10E,12Z-octadecadienoic-9,10,12,13-d4 acid
12-HETE-d8	12(S)-HETE-d8	12S-hydroxy-5Z,8Z,10E,14Z-eicosatetraenoic-5,6,8,9,11,12,14,15-d8 acid
5-HETE-d8	5(S)-HETE-d8	5S-hydroxy-6E,8Z,11Z,14Z-eicosatetraenoic-5,6,8,9,11,12,14,15-d8 acid
14,15-EET-d11	(±)11(12)-EET-d11	(±)11(12)-epoxy-5Z,8Z,14Z-eicosatrienoic-16,16,17,17,18,18,19,19,20,20,20 acid
5-oxo-ETE-d7	5-OxoETE-d7	5-oxo-6E,8Z,11Z,14Z-eicosatetraenoic-6,8,9,11,12,14,15-d7 acid
11,12-EET-d11	(±)11(12)-EET-d11	(±)11(12)-epoxy-5Z,8Z,14Z-eicosatrienoic-16,16,17,17,18,18,19,19,20,20,20 acid
8,9-EET-d11	(±)8(9)-EET-d11	(±)8(9)-epoxy-5Z,8Z,14Z-eicosatrienoic-16,16,17,17,18,18,19,19,20,20,20 acid

Table S1 continued

5,6-EET-d11	(±)5(6)-EET-d11	(±)5(6)-epoxy-8Z,11Z,14Z-eicosatrienoic-16,16,17,17,18,18,19,19,20,20,20-d11 acid
EPA-d5	Eicosapentaenoic Acid-d5	5Z,8Z,11Z,14Z,17Z-eicosapentaenoic-19,19,20,20,20-d5 acid
DHA-d5	Docosahexaenoic Acid-d5	4Z,7Z,10Z,13Z,16Z,19Z-docosahexaenoic-21,21,22,22,22-d5 acid
AA-d8	Arachidonic Acid-d8	5Z,8Z,11Z,14Z-eicosatetraenoic-5,6,8,9,11,12,14,15-d8 acid

Table S2. Lipid mediators measured in the study.

Abbreviation	Full name/Synonym	Systematic name
$PGF_{1\alpha}$	Prostaglandin F1α	9S,11R,15S-trihydroxy-13E-prostaenoic acid
6-keto-$PGF_{1\alpha}$	6-keto-Prostaglandin F1α	6-oxo-9S,11R,15S-trihydroxy-13E-prostenoic acid
6,15dkdh-$PGF_{1\alpha}$	6,15-diketo-13,14-dihydro-PGF1a	6,15-dioxo-9S,11R-dihydroxyprostanoic acid
$PGF_{2\alpha}$	Prostaglandin F2α	9S,11R,15S-trihydroxy-5Z,13E-prostadienoic acid
5-iso-$PGF_{2\alpha}$-VI	Isoprostane F2α-I	5,9S,11R-trihydroxy-6E,14Z-prostadienoic acid-cyclo[8S,12R]
PGE_2	Prostaglandin E2	9-oxo-11R,15S-dihydroxy-5Z,13E-prostadienoic acid
PGD_2	Prostaglandin D2	9S,15S-dihydroxy-11-oxo-5Z,13E-prostadienoic acid
TXB_2	Thromboxane B2	9S,11,15S-trihydroxy-thromboxa-5Z,13E-dien-1-oic acid
12-HHT	12S-HHTrE	12S-hydroxy-5Z,8E,10E-heptadecatrienoic acid
11-HETE	11-HETE	11-hydroxy-5Z,8Z,11E,14Z-eicosatetraenoic acid
12-HETE	12-HETE	12-hydroxy-5Z,8Z,10E,14Z-eicosatetraenoic acid
13-HODE	13S-HODE	13S-hydroxy-9Z,11E-octadecadienoic acid
LTC_4	Leukotriene C4	5S-hydroxy,6R-(S-glutathionyl),7E,9E,11Z,14Z-eicosatetraenoic acid
LTE_4	Leukotriene E4	5S-hydroxy,6R-(S-cysteinyl),7E,9E,11Z,14Z-eicosatetraenoic acid
5,15-diHETE	5,15-diHETE	(5Z,9E,11Z,13E)-8,15-dihydroxyicosa-5,9,11,13-tetraenoic acid
12,13di-HOME	12,13-DiHOME	12,13-dihydroxy-9Z-octadecenoic acid
tetranor-12-HETE	Tetranor-12R-HETE	8R-hydroxy-4Z,6E,10Z-hexadecatrienoic acid
9,10di-HOME	Leukotoxin diol	9,10-dihydroxy-12Z-octadecenoic acid
14,15-DHET	14,15-DiHETE	(+/-)-14,15-dihydroxy-5Z,8Z,11Z,17Z-eicosatetraenoic acid
11,12-DHET	11,12-DiHETE	(+/-)-11,12-dihydroxy-5Z,8Z,14Z,17Z-eicosatetraenoic acid
18-HEPE	(+/-)-18-HEPE	(+/-)-18-hydroxy-5Z,8Z,11Z,14Z,16E-eicosapentaenoic acid
12-HEPE	(+/-)-12-HEPE	(+/-)-12-hydroxy-5Z,8Z,10E,14Z,17Z-eicosapentaenoic acid
18-HETE	18-HETE	18-hydroxy-5Z,8Z,11Z,14Z-eicosatetraenoic acid
5-HEPE	(+/-)-5-HEPE	(+/-)-5-hydroxy-6E,8Z,11Z,14Z,17Z-eicosapentaenoic acid
14-HDoHE	(+/-)-14-HDoHE	(+/-)-14-hydroxy-4Z,7Z,10Z,12E,16Z,19Z-docosahexaenoic acid
15-HETE	15-HETE	15-hydroxy-5Z,8Z,11Z,13E-eicosatetraenoic acid
17-HDoHE	(+/-)-17-HDoHE	(+/-)-17-hydroxy-4Z,7Z,10Z,13Z,15E,19Z-docosahexaenoic acid
9-HODE	9-hydroxy-trans-10,cis-12-octadecadienoic acid	9-hydroxy-10E,12Z-octadecadienoic acid
9HpODE	9S-HpODE	9S-hydroperoxy-10E,12Z-octadecadienoic acid
12oxo-ETE	12-oxo-ETE	12-oxo-5Z,8Z,10E,14Z-eicosatetraenoic acid
15oxo-ETE	15-Oxo-ETE	15-oxo-5Z,8Z,11Z,13E-eicosatetraenoic acid
7-HDoHE	(+/-)-17-HDoHE	(+/-)-17-hydroxy-4Z,7Z,10Z,13Z,15E,19Z-docosahexaenoic acid
8-HETE	8-HETE	8-hydroxy-5Z,9E,11Z,14Z-eicosatetraenoic acid

Table S2 continued

9-HETE	9-HETE	9-hydroxy-5Z,7E,11Z,14Z-eicosatetraenoic acid
4-HDoHE	(+/-)-14-HDoHE	(+/-)-14-hydroxy-4Z,7Z,10Z,12E,16Z,19Z-docosahexaenoic acid
5-HETE	5-HETE	5-hydroxy-6E,8Z,11Z,14Z-eicosatetraenoic acid
12(13)-EpOME	Vernolic acid	(+/-)-12(13)-epoxy-9Z-octadecenoic acid
5oxo-ETE	5-Oxo-ETE	5-oxo-6E,8Z,11Z,14Z-eicosatetraenoic acid
11,12-EET	(+/-)11,12-EpETrE	11,12-epoxy-5Z,8Z,14Z-eicosatrienoic acid

Table S3. Lipid mediators below the detection limit.

Abbreviation	Synonym	Systematic name
20cooh-LTB4	20-carboxy LTB_4	5S,12R-dihydroxy-6Z,8E,10E,14Z-eicosatetraene-1,20-dioic acid
RvE1	Resolvin E1	5S,12R,18R-trihydroxy-6Z,8E,10E,14Z,16E-eicosapentaenoic acid
20-oh-LTB4	20-Hydroxy-leukotriene B4	(5S,6Z,8E,10E,12R,14Z)-5,12,20-trihydroxyicosa-6,8,10,14-tetraenoic acid
TXB3	Thromboxane B3	9S,11,15S-trihydroxy-thromboxa-5Z,13E,17Z-trien-1-oic acid
PGF3α	Prostaglandin F3α	9S,11R,15S-trihydroxy-5Z,13E,17Z-prostatrienoic acid
PGF2β	Prostaglandin F2β	9R,11R,15S-trihydroxy-5Z,13E-prostadienoic acid
LXA5	Lipoxin A5	5S,6R,15S-trihydroxy-7E,9E,11Z,13E,17Z-eicosapentaenoic acid
PGE3	Prostaglandin E3	9-oxo-11R,15S-dihydroxy-5Z,13E,17Z-prostatrienoic acid
PGD3	Prostaglandin D3	9S,15S-dihydroxy-11-oxo-5Z,13E,17Z-prostatrienoic acid
LTD4	Leukotriene D4	5S-hydroxy-6R-(S-cysteinylglycinyl)-7E,9E,11E,14Z-eicosatetraenoic acid
RvD2	Resolvin D2	7S,16R,17R-trihydroxy-4Z,8E,10Z,12E,14E,19Z-docosahexaenoic acid
14,15-LTC4	14,15-Leukotriene C4	15S-hydroxy,14R-(S-glutathionyl)-5Z,8Z,10E,12E-eicosatetraenoic acid
LXA4	Lipoxin A4	5S,6R,15S-trihydroxy-7E,9E,11Z,13E-eicosatetraenoic acid
RvD1	Resolvin D1	7S,8R,17S-trihydroxy-4Z,9E,11E,13Z,15E,19Z-docosahexaenoic acid
PGA2	Prostaglandin A2	9-oxo-15S-hydroxy-5Z,10Z,13E-prostatrienoic acid
8,15-DiHETE	8,15-DiHETE	(5Z,9E,11Z,13E)-8,15-dihydroxyicosa-5,9,11,13-tetraenoic acid
NPD1	Neuroprotectin D1	10R,17S-dihydroxy-4Z,7Z,11E,13E,15Z,19Z-docosahexaenoic acid
LTB4	Leukotriene B4	5S,12R-dihydroxy-6Z,8E,10E,14Z-eicosatetraenoic acid
11,12-DHET	(±)11,12-DiHETrE	(±)11,12-dihydroxy-5Z,8Z,14Z-eicosatrienoic acid
9-HEPE	(+/-)-9-HEPE	(+/-)-9-hydroxy-5Z,7E,11Z,14Z,17Z-eicosapentaenoic acid
11-HEPE	(+/-)-11-HEPE	(+/-)-11-hydroxy-5Z,8Z,12E,14Z,17Z-eicosapentaenoic acid
20-HETE	20-hydroxy Arachidonic Acid	20-hydroxy-5Z,8Z,11Z,14Z-eicosatetraenoic acid
13-OxoODE	13-OxoODE	13-oxo-9,11-octadecadienoic acid
10-HDoHE	(+/-)-10-HDoHE	(+/-)-10-hydroxy-4Z,7Z,11E,13Z,16Z,19Z-docosahexaenoic acid
17-keto-DPA	17-oxo-DPA	7Z,10Z,13Z,15E,19Z-17-keto-docosapentaenoic acid

Table S4. Primers used in the study.

Gene	Forward primer, 5'-	Reverse primer, 5'-
Acadm	AGTATGCCCTGGATAGGAAGACAT	CTTGGTGCTCCACTAGCAGCT
Acox1	GGGTCATGGAACTCATCTTCGA	GAATGAACTCTTGGGTCTTGGG
Adipoq	GATGGCAGAGATGGCACTCC	CTTGCCAGTGCTGCCGTCAT
Arg1	GTCATTTGGGTGGATGCTCAC	TCCTGGTACATCTGGGAACTTTC
Ccl2	GTGTTGGCTCAGCCAGATGC	GCTTGGTGACAAAAACTACAGC
Cebpa	CAAGAACAGCAACGAGTACCG	GTCACTGGTCAACTCCAGCAC
Cebpb	GGGTTTCGGGACTTGATGC	ACATCAACAACCCCGCAGG
CIDEA	GACAGAAATGGACACCGGG	GATTCCTTAACACGGCC
Cpt1a	TACTGCTGTATCGTCGCACG	GACGAATAGGTTTGAGTTCCTCAC
Dio2	CAGTGTGGTGCACGTCTCCAATC	TGAACCAAAGTTGACCACCAG
Fabp4	TGGAAGCTTGTCTCCAGTGA	AATCCCCATTTACGCTGATG
Fizz1	CCATAGAGATTATCGTGGAG	TGGTCCTGTCAACGAGTAAG
G6pc	ACCGGACCAGGAAGTCCC	GCAATGCCTGACAAGACTCC
Gyk	CAAATGCAAGCAGGACGATG	GGCCCCAGCTTTCATTAGG
Il13	CAGCCTCCCCGATACCAAAAT	GCGAAACAGTTGCTTTGTGTAG
Il33	CCTGCCTCCCTGAGTACATACA	CTTCTTCCCATCCACACCGT
Il4	GGTCTCAACCCCCAGCTAGT	GCCGATGATCTCTCTCAAGTGAT
Il5	TCAGGGGCTAGACATACTGAAG	CCAAGGAACTCTTGCAGGTAAT
Klf5	GCTGGTCCAGACAAGATGTG	GGAAGACGTTCATGTTGATGC
Nos2	CTACGCCTTCAACACCAAGG	CAAACAAGCATACCTGAAGG
Pck1	GTGCCTGTGGGAAGACTAAC	CCTTAAGTTGCCTTGGGCAT
Pparg1	GTGTGACAGACAAGATTTGAAAG	GCTTGATGTCAAAGGAATGCG
Pparg2	ACAGCAAATCTCTGTTTTATGC	TGCTGGAGAAATCAACTGTGG
Ppargc1a	CATTTGATGCACTGACAGATGGA	CCGTCAGGCATGGAGGAA
Prdm16	CAGCACGGTGAAGCCATTC	GCGTGCATCCGCTTGTG
Pref1	CGAAATAGACGTTCGGGCTT	TCGTACTGGCCTTTCTCCAG
Ptgs1	TTACTATCCGTGCCAGAACCA	CCCGTGCGAGTACAATCACA
Ptgs2	GACTGGGCCATGGAGTGG	CACCTCTCCACCAATGACC
Siglecf	CTGGCTACGGACGGTTATTCG	GGAATTGGGGTACTGGACTTG
Slc2a4	CAGAAGGTGATTGAACAGAGC	CCCTGATGTTAGCCCTGAG
Tbp	ACCCTTCACCAATGACTCCTATG	ATGATGACTGCAGCAAATCGC
Tnfa	CCCTCACACTCAGATCATCTTCT	GCTACGACGTGGGCTACAG
Ucp1	AGCCGGCTTAATGACTGGAG	TCTGTAGGCTGCCCAATGAAC
